# Brain transcriptome profiles in mouse model simulating features of post-traumatic stress disorder

**DOI:** 10.1186/s13041-015-0104-3

**Published:** 2015-02-28

**Authors:** Seid Muhie, Aarti Gautam, James Meyerhoff, Nabarun Chakraborty, Rasha Hammamieh, Marti Jett

**Affiliations:** Advanced Biomedical Computing Center, Frederick National Lab for Cancer Research, Fort Detrick, MD 21702 USA; Integrative Systems Biology Program, U.S. Army Center for Environmental Health Research, 568 Doughten Drive, Fort Detrick, MD 21702-5010 USA

**Keywords:** Social stress, Mouse model, PTSD, Aggressor exposure, Microarray, Fear response

## Abstract

**Background:**

Social-stress mouse model, based on the resident-intruder paradigm was used to simulate features of human post-traumatic stress disorder (PTSD). The model involved exposure of an intruder (subject) mouse to a resident aggressor mouse followed by exposure to trauma reminders with rest periods. C57BL/6 mice exposed to SJL aggressor mice exhibited behaviors suggested as PTSD-in-mouse phenotypes: intermittent freezing, reduced locomotion, avoidance of the aggressor-associated cue and apparent startled jumping. Brain tissues (amygdala, hippocampus, medial prefrontal cortex, septal region, corpus striatum and ventral striatum) from subject (aggressor exposed: Agg-E) and control C57BL/6 mice were collected at one, 10 and 42 days post aggressor exposure sessions. Transcripts in these brain regions were assayed using Agilent’s mouse genome-wide arrays.

**Results:**

Pathways and biological processes associated with differentially regulated genes were mainly those thought to be involved in fear-related behavioral responses and neuronal signaling. Expression-based assessments of activation patterns showed increased activations of pathways related to anxiety disorders (hyperactivity and fear responses), impaired cognition, mood disorders, circadian rhythm disruption, and impaired territorial and aggressive behaviors. In amygdala, activations of these pathways were more pronounced at earlier time-points, with some attenuation after longer rest periods. In hippocampus and medial prefrontal cortex, activation patterns were observed at later time points. Signaling pathways associated with PTSD-comorbid conditions, such as diabetes, metabolic disorder, inflammation and cardiac infarction, were also significantly enriched. In contrast, signaling processes related to neurogenesis and synaptic plasticity were inhibited.

**Conclusions:**

Our data suggests activations of behavioral responses associated with anxiety disorders as well as inhibition of neuronal signaling pathways important for neurogenesis, cognition and extinction of fear memory. These pathways along with comorbid-related signaling pathways indicate the pervasive and multisystem effects of aggressor exposure in mice, potentially mirroring the pathologic conditions of PTSD patients.

**Electronic supplementary material:**

The online version of this article (doi:10.1186/s13041-015-0104-3) contains supplementary material, which is available to authorized users.

## Background

Post-traumatic stress disorder (PTSD) is an anxiety disorder, which can result from exposure to a traumatic event(s). PTSD can manifest itself in a number of ways such as a persistent re-experiencing of the traumatic event, arousal, avoidance, numbing, fear, and/or startle responses [1]. PTSD patients may also exhibit impaired cognition, working memory deficit, mood disorders and diminished social activities [2]. Common PTSD co-morbid conditions include depression, obesity, diabetes, peripheral inflammation, cardiovascular problems, and metabolic disorders [3].

Anxiety disorders, including PTSD, encompass heterogeneous conditions that are associated with abnormalities in the fear response circuitry in brain structures such as the medial prefrontal cortex, amygdala, hippocampus, and nucleus accumbens (Additional file 1: Figure S1). Changes in the activity and functional connectivity of these brain regions lead to abnormalities in the perception and interpretation of traumatic events and to the development and maintenance of anxiety disorders [4-7]. For example, the hippocampus (HC) is implicated in contextual fear learning, in trauma memory consolidation, and retrieval (intrusive re-experiencing of the traumatic event); the amygdala (AY) is associated with cue conditioning, hyper-vigilance, heightened arousal, learning, and expressing fear behavior [8,9]; the medial prefrontal cortex (MPFC) is involved in cognition, emotional regulation, and fear extinction; and the nucleus accumbens (NAc, part of the ventral striatum, VS) is associated with reward-related behavioral abnormalities such as depression [10]. Other brain regions, such as corpus striatum (ST) and septal region (SE), are implicated in processing and responding to reward and aversive stimuli, lack of motivation, and recall of intrusive traumatic cues in the form of flashbacks [11,12].

Impaired extinction of fear-potentiated startle and enhanced cue conditioning in these brain regions (of traumatized patients and animal models) can be manifested in the forms of hypervigilance, arousal, and avoidance symptoms [13]. In particular, failure to inhibit fear learning and fear memory consolidation are considered to be precipitating factors in the development of PTSD [14]. With regards to cognition, PTSD patients showed significantly less activation in sensory association areas, suggesting diversion of attention from the presented stimuli, perhaps due to increased focus on the elicited trauma memory [15-17]. It has been suggested that cognitive impairments exhibited by people with PTSD result from intrusive flashback memories that transiently interfere with ongoing cognitive processing [17].

Molecular and cellular encoding of traumatic events and behavioral responses are presumed to be reflected in synaptic plasticity or changes in the activity and functional connectivity among AY, HC and mPFC [18-21]. Specifically, changes in signaling molecules associated with synaptic transmission and plasticity are implicated as a primary substrate for fear learning and memory, thus putatively leading to PTSD in human and to behavioral features of PTSD in animals. This could occur because the expression levels and types of signaling molecules determine spike-timing-dependent plasticity in relation to traumatic perceptions and responses. This could lead to either a long-term synaptic potentiation (LTP) increase in synaptic strength and increase in excitatory post-synaptic potential, potentially enhancing the manifestation of traumatic disorder, or to long-term synaptic depression (LTD) leading to a decrease in synaptic strength and a decrease in EPSP size, suppressesing fear extinction processes [22,23]. Understanding the molecular underpinnings of fear learning and memory following trauma exposure or presentation of an aversive stimulus would be critical to identifying prognostic biomarkers and for developing an intervention to apply post-trauma, before the onset of pathological symptoms.

Subsequent to trauma exposure, the symptoms of stress reaction typically develop over varying amounts of time (typically from days to months), resulting in heterogeneous pathologies. The heterogeneity of PTSD symptoms suggests that its etiology is diverse, and there are still no known or accepted molecular biomarkers for diagnosis of PTSD. Current pharmacotherapies for PTSD are applicable after symptoms manifest, and primarily consist of selective serotonin reuptake inhibitors. There are no FDA-approved pharmacological interventions available for the treatment of traumatized individuals to forestall the development of PTSD prior to the onset of pathological symptoms. Many factors hinder advances towards identifying prognostic biomarkers and potential pre-symptomatic therapeutic targets for PTSD. One of these factors, despite great advances in brain imaging, is a lack of real time visualization of cellular reactions in specific brains regions of individuals at risk for PTSD and patients with the disorder. Hence animal models are required to obtain appropriate brain regions for genomic, genetic and other high throughput molecular studies.

Increasing evidence suggests that precipitating factors such as chronic stress (including social defeat stress) induce changes in the functional connectivity within the fear circuitry, and such changes mediate trauma-related behavior alerations in animal models of anxiety disorders [24,25]. Such behavioral responses include startle response, hyperactivity, avoidance, freezing, grooming, rearing, and deficiencies in novel object recognition and in territorial behavior. The molecular and cellular origins of these behavioral abnormalities have been suggested to include changes in glutamatergic and GABAergic synaptic plasticity, dopamine neuron excitability, epigenetic and transcriptional mechanisms, and neurotrophic factors [26-28]. However, the genome-wide molecular basis for the interplay of different behaviors in anxiety disorders is not very clear. We hypothesize that chronic exposure to stress alters gene expression patterns which are important for the functional activity, connectivity and signaling among neurons of these brain regions, thus leading to the development of anxiety and depression-like behaviors.

We and others have shown that aggressor exposed (Agg-E) social-stress mouse models elicit behaviors such as submissive posture, freezing during locomotion, vertical rearing, grooming, aggressor barrier avoidance, hyperactivity, jumping, and impaired cognition (deficiency in novel object recognition) [29,30]. These might be considered to be PTSD-like phenotypes.

In this study, our objective was to correlate our previous behavioral observations [29] with changes at the molecular level in brain regions critical for fear learning, fear memory consolidation/extinction and fear response/expression. Here we attempted to understand how environmental stimuli (exposure to aggressor) and transcriptome interact and influence each other in the context of behaviors suggesting fear. Such approaches are integral to our understanding and treatment of stress-related disorders such as PTSD (interaction of genes, brain, and behavior). Although a candidate gene approach, in conjunction with endophenotyping, would be important to solve the puzzle of PTSD etiology, genome-wide screening for transcriptome changes has the potential to lead to new candidate targets and pathways specific to stress disorders, and to facilitate or hasten the one gene or few candidate genes approaches. It may also reveal new molecular mechanisms and connections, which may not be apparent from a single gene or genotyping approach.

Toward this goal, we performed global gene expression profiling of different brain regions implicated in fear and anxiety processing. Genome-wide transcriptome changes were assessed in brain regions collected from male C57BL/6 mice exposed to male SJL aggressor mice. The behaviors elicited suggest a possible PTSD-in-mouse phenotype: immobility, avoidance of aggressor-associated stimuli (aggressor odor), jumping (indicating hyperactivity), freezing during locomotion (suggesting fear), and reduced locomotion. Transcriptome alterations in six brain regions: HC, AY, MPFC, VS (contains NAc), SE, and ST (Additional file 1: Figure S1) from Agg-E mice were measured using Agilent’s mouse genome-wide arrays to identify transcripts and associated pathways that reveal potential molecular mechanisms of stress disorders. Profiles of genome-wide transcriptome changes were carried out at different time points to assess how the time course dynamics of transcripts indicate molecular events associated with traumatic fear learning and memory along the developmental trajectories of traumatic-anxiety disorders.

We identified a number of differentially expressed genes (DEGs) across different brain regions and time points. Functional and pathway analyses of DEGs suggested possible roles in anxiety-related behavioral responses, synaptic plasticity, neurogenesis, inflammation, obesity (metabolic disorder), and cardiac infarction. In particular, transcripts important for the synthesis of neurotransmitters, generation and development of the hippocampus, axonogenesis, dendritic branching, splice variant processing, and dopaminergic and serotonergic pathways were affected.

## Results

### Behavioral evaluation of mouse model

We established [29] a rodent model manifesting PTSD-like behavioral features. We believe that, because the stressor depends on antagonistic social interaction within a conspecific, this model offers potential advantages over the brief foot-shock model in simulating traumatic events of conflict-like situations. Especially as both the stressor and the behaviors assessed are essentially within the social realm. Concerns [31] that the repeated social stress model may allow the animals to habituate rather than triggering the PTSD-like features was countered by implementing the ‘randomness’ in the occurrence of the life-threatening conditions. Thereby we incorporated a critical dimension of traumatic psychosocial stress with regard to the “ethological validity” of the unpredictable and uncontrollable nature of the PTSD simulating trauma. The hypothesis was further validated as we found the mice displaying many traumatic features after a long time interval. The ‘habituation’ related concern was further mitigated as the stressed mice displayed significantly reduced urine markings, a signature of inhibited territorial behavior in the course of 10-day stress session. Territorial urine markings of Agg-E mice were three-fold lower than those of control mice with linear regression significance of p = 0.02 [29]. Post 10-day Agg-E, stressed mice maintained decreased but not significant urine marking (p = 0.07) compared to the controls. During the same time course, we observed an increased bodyweights of Agg-E mice. Linear regression model showed significant difference (p < 0.001) in gain of body weights by the stressed cohorts. In addition, Agg-E groups showed significantly increased temperature (*p* = 0.006), and 30% decreased but not statistically significant corticosterone levels ((p = 0.06).

In addition, the present model supports a set of predefined validity criteria for an animal model of PTSD [31,32]. For instance, the “face validity” of symptoms simulating PTSD-like features was manifested by introducing the stressed mice to the contextual cues immediately after withdrawing the trauma as well as at a delayed interval or rest period (the latter being equivalent to nearly three years of human life). We observed a significant display in associative fear memory, anxiety and hypo- and hyper-responses immediately after the trauma-withdrawal; and many of these behavioral traits, such as the restrained tail rattling (an agonistic behavior, hypo-response) and grooming (an anxiety-like feature, hyper-response) sustained through the delayed interval. Specifically, while the number of control animals displaying tail rattling gradually increased with the passage of 42 days, none of the stressed mice displayed tail rattling even 42 days after the end of stress exposure. Moreover, the duration of grooming displayed by the stressed mice remained 2.5 fold higher (p < 0.05) than the controls, after 42 days post-10 days of Agg-E. Likewise, freezing, a hypo-active fear response remained significant from day 1 (p < 0.0001) to 28 days (p < 0.05) post-10 day stress. Suppressing a natural instinct namely the vigilance of novel object, the stressed animals, showed significant avoidance of the aggressor’s partition (p < 0.0001) 1 day after stress; however Agg-E mice exhibited decreased avoidance at 42 days of rest (delayed) period post 10 day exposure session.

The present model attested an essential criterion of PTSD namely the persistence (or re-emergence) of a contextual fear memory. We further noticed a ‘dose-response’ in displaying PTSD feature, as in comparing the 5-day and 10-day long traumatic burdens, the later proved more deleterious. The “construct validity” of the rodent PTSD model representing the cellular and molecular processes was underlined by the atypical increase of many blood cells’ load, previously associated with stress [33]. Significant myocardial atrophies caused by this model persisted after the delayed interval, which potentially implied the cardiac damages caused by PTSD primarily on the war veterans. Additional histological analysis showed lack of the expected increase in dendritic spine density of pyramidal neurons in MPFC (medial prefrontal cortex, an essential brain region for fear memory extinction and memory sorting). Together this model strategically modified the social stress model to reasonably simulate aspects of combat-related PTSD, and validated some of the essential criteria of PTSD in rodent-models.

### Gene expression analyses of brain regions

Feature-extracted and quantile normalized microarray data from brain regions were analyzed to assess stress-affected biological processes and pathways in Agg-E mice as compared to controls.

**Figure 1:**
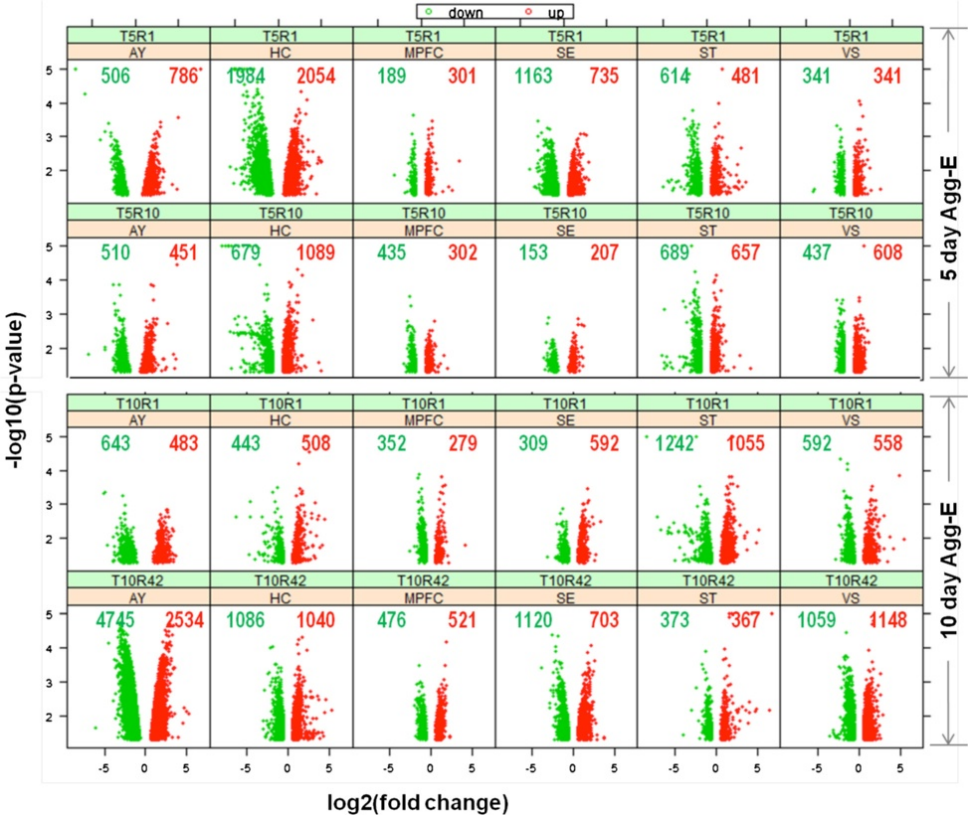
**Differentially expressed genes (DEGs) across brain regions at different time points (T5R1, T5R10, T10R1 and T10R42).** The scatter plots show both log_2_ -fold changes and negative log_10_ *p*-values in six brain regions (across) and four time-points (down); the numbers of up- and down-regulated transcripts in each group are also shown. Key: amygdala (AY), hippocampus (HC), medial prefrontal cortex (MPFC), septal region (SE), corpus striatum (ST), and ventral striatum (VS), T: number of days of trauma or aggressor exposure; R: post-trauma tissue collection days; Agg-E: aggressor-exposed.

Differentially expressed genes (DEGs), compared between Agg-E and control (C-ctrl) mice for each brain region (AY, HC, MPFC, SE, ST and VS), were identified at four different time points (T5R1, T5R10, T10R1 and T10R42) (Table 1) using Moderated T-test, *p* ≤ 0.05 (Figure 1). For the 10-day Agg-E groups, there were more DEGs after the longer rest period (T10R42) except for ST which showed a large decrease in DEGs as compared to the T10R1 group. The numbers of DEGs of 5-day Agg-E groups were greater at T5R1 for AY, HC and SE regions; at T5R10, the numbers of DEGs increased for MPFC, ST and VS, while by contrast the numbers were greatly decreased for HC and SE.

**Table 1:** **Summary of experimental conditions and collected tissues**

**Study group (time points)**	**Experimental and control groups (5 mice/group)**	*Subject mouse strain	**Aggressor strain**	**Exposure length (days)**	**Post Agg-E length (days)**
T5R1	Agg-E_T5R1	C57BL/6	SJL	5	1
	C-ctr_5R1	C57BL/6	SJL	5	1
T5R10	Agg_E_T5R10	C57BL/6	SJL	5	10
	C-ctr_T5R10	C57BL/6	SJL	5	10
T10R1	Agg-E_T10R1	C57BL/6	SJL	10	1
	C-ctr_T10R1	C57BL/6	SJL	10	1
T10R42	Agg-E_T10R42	C57BL/6	SJL	10	42
	C-ctr_T10R42	C57BL/6	SJL	10	42

*collected brain regions: HC: hippocampus, AY: amygdala, MPFC: medial prefrontal cortex, VS: ventral striatum or nucleus accumbens, SE: septal region, ST: corpus striatum.

The greater divergence between 10-day Agg-E and C-ctrl groups after 42 days of rest may be due to either of two factors. One factor may be due to the faster recovery of the C-ctrl mice from handling, confinement and hunger stressors compared to the rate of recovery of the Agg-E groups. Another factor may be that multiple processes leading to a return to homeostasis versus further consolidation of PTSD-like disorders (or combination of both) may involve expression of many more and different sets of genes in the Agg-E mice.

### Functional and pathway enrichments and visualization of associated networks

**Figure 2:**
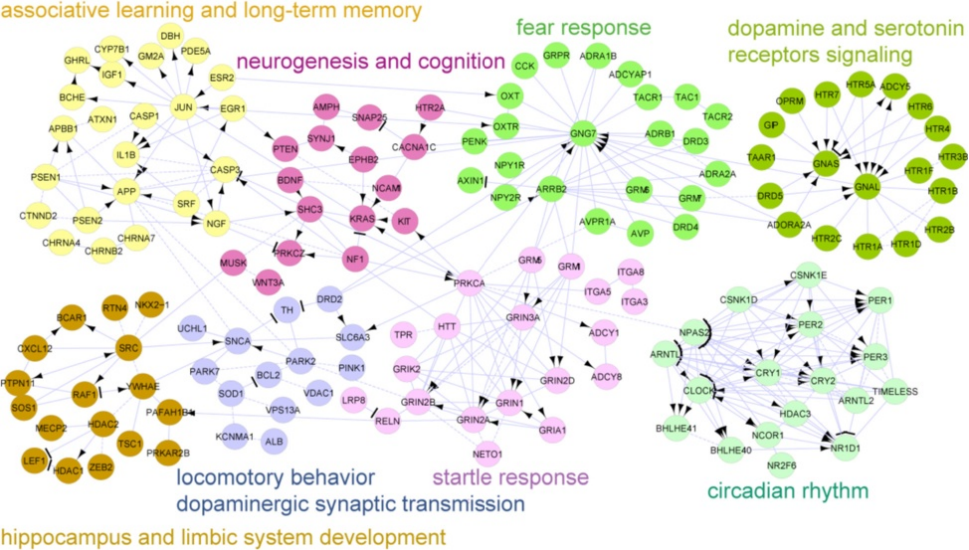
**Modular networks of DEGs associated with circadian clock, behavioral response and synaptic transmission; each module (nodes of the same color) forms a functional module.**

Functional and pathway enrichments of DEGs of the different brain regions led to the identification of modular-networks (functional interaction networks) consisting of genes implicated in anxiety-related behavioral responses and the underlying synaptic processes (Figure 2). Each module of this network has many more member DEGs, which were excluded for clarity (visualization) to show only representative members. Also, the list of pathway and functional modules identified were longer than shown in Figure 2 (Additional file 2: Table S1). Member DEGs involved in long-term memory, associative learning, and limbic system development were largely from the HC; DEGs associated with startle and fear responses were largely from the AY; and DEGs associated with circadian rhythm, cognition, neurogenesis, locomotory behavior, dopaminergic and serotonergic pathways were from different brain regions.

**Figure 3:**
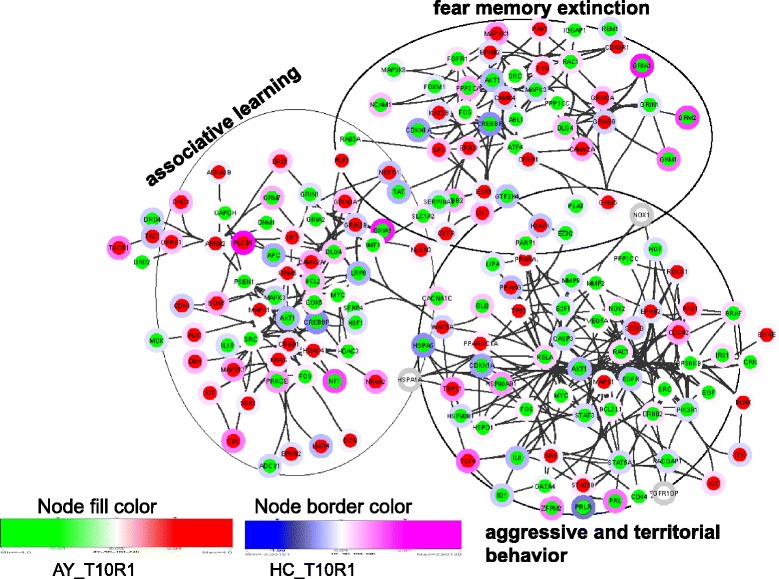
**Networks of DEGs involved in associative learning, fear memory extinction, and aggressive and territorial behaviors.** Nodes are colored using AY_T10R1 and HC_T10R1.

**Figure 4:**
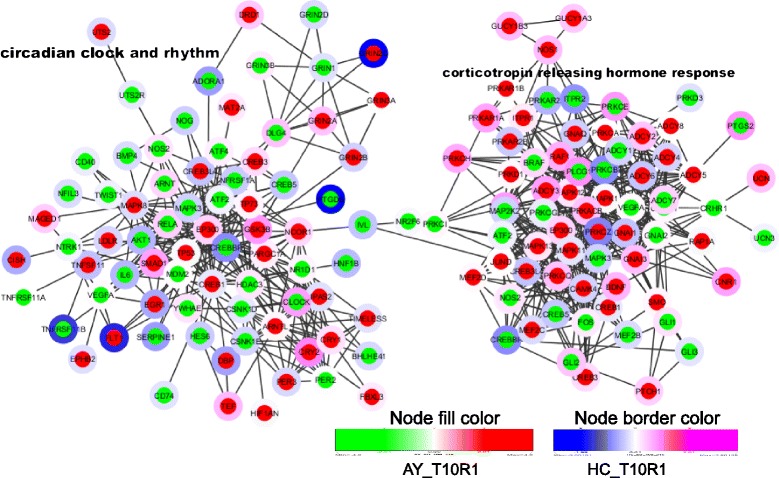
**Networks of DEGs involved in circadian rhythm and corticotropin releasing factor signaling.** Nodes are colored using AY_T10R1 and HC_T10R1.

Separation of each module, adding more member DEGs, and coloring nodes based on their expression level in HC and AY for the one-day post 10-day Agg-E group (T10R1), showed how a particular behavioral response or biological process behaves in these two important brain regions (Figures 3 and 4). For example, modular network DEGs involved in associative learning were largely induced in AY, whereas DEGs associated with aggressive and territorial behaviors were largely suppressed in AY, suggesting involvement in impaired territorial and aggressive behavior (Figure 3). This inference is also corroborated by the finding of significant reduction of urine marking. Many of the DEGs associated with circadian rhythm were suppressed in AY, suggesting a circadian rhythm disruption, whereas those involved in corticotrophin releasing hormone signaling were largely induced in AY (Figure 4), indicating aggression (trauma) potentiated hypothalamic-pituitary-adrenal (HPA)-axis signaling, thus leading to anxiety-related behavioral responses. The expression patterns of some of the important nodes showed different directions in AY and HC. The differential responses between these two important brain regions are consistent with suboptimal communication between the putative fear response center (AY) and the center associated with contextual processing (HC). Examining fewer member genes among those associated with some of stress-induced processes and pathways such as glucocorticoid receptor signaling, neurotransmitter secretion, inflammation and growth factor receptors, we still see a mix of same and opposing directions of expression of these genes between AY and HC (Additional file 1: Figure S2). Some of the DEGs involved in social withdrawal, immobility, long-term memory, and startle, anxiety and fear responses were down-regulated in AY (Additional file 1: Figure S3). Many of these down-regulated genes were also found to be associated with neurotrophic factors signaling, fear extinction, and functions related to emotional regulation.

**Figure 5:**
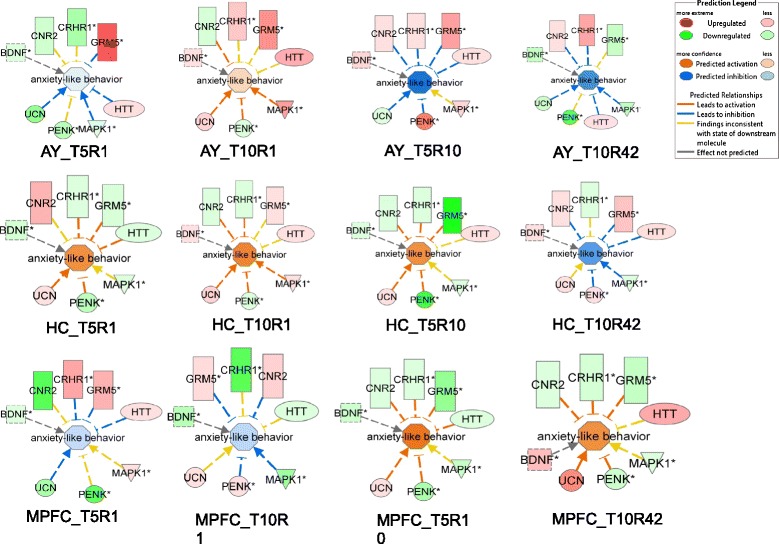
**Activation pattern of anxiety-like behavior in amygdala (AY), hippocampus (HC) and medial prefrontal cortex (MPFC) and expression directions of associated genes.**

As suppressed or induced expression of associated genes does not necessarily correspond to the activation or inhibition state of the corresponding behavioral responses, we calculated an activation z-score for each significantly enriched behavior and biological process based on the expression values of associated DEGs in each brain region at each time point. This approach is important to further identify which behavioral responses and biological processes may have been activated or inhibited. For example, Figure 5 shows the activation pattern of anxiety-like behavior in AY, HC and MPFC at both early (T5R1, T10R1) and late (T5R10, T10R42) rest periods. Here, it is more obvious how the expression direction of each DEG contributes to the predicted activation (red central node) or inhibition (blue central node) of the anxiety-like behavior (Figure 5). The pattern became clearer not only within a single network but also in comparison among different brain regions and time points. At T10R1, genes associated with anxiety-like behavior are activated in AY but inhibited in the MPFC, which is consistent with reported observations of exaggerated activation of AY and delayed regulation by MPFC during early stage amygdala-dependent fear responses [34,35]. On the other hand, at longer rest periods (T5R10, T10R42), the opposite pattern of activation occurred between AY and MPFC (Figure 5), which may indicate that the attenuation of the anxiety-like response in AY was probably due to emotional regulation by MPFC. Anxiety-like behavior also showed activation or attenuation at earlier and at later rest periods, respectively, in AY (Additional file 1: Figure S4). Activation z-scores for fear conditioning and startle response in AY, HC and MPFC also showed different activation or attenuation patterns across time points and regions (Additional file 1: Figures S5 and S6).

**Figure 6:**
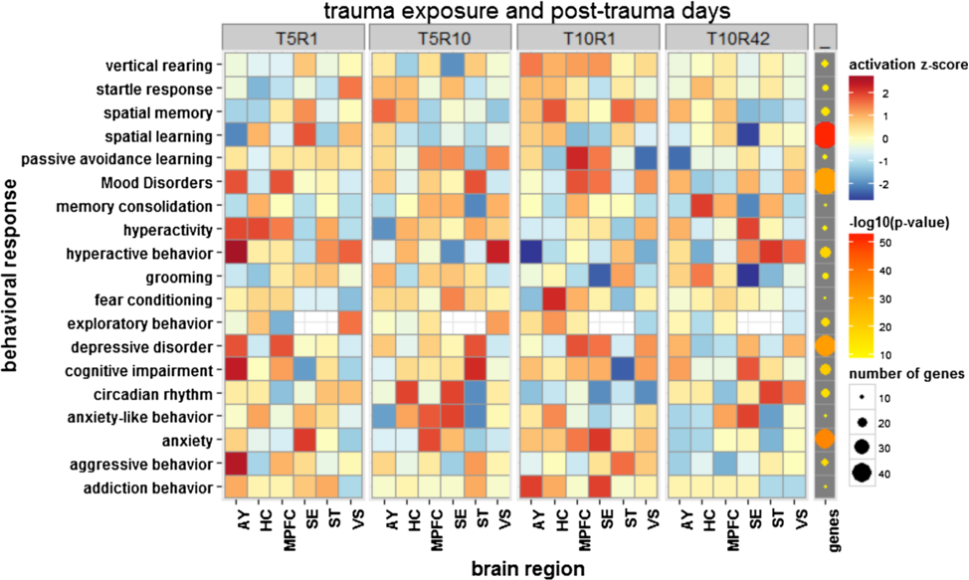
**Behavioral response and neurological disorders associated with DEGs of brain regions at four different time points.**

**Figure 7:**
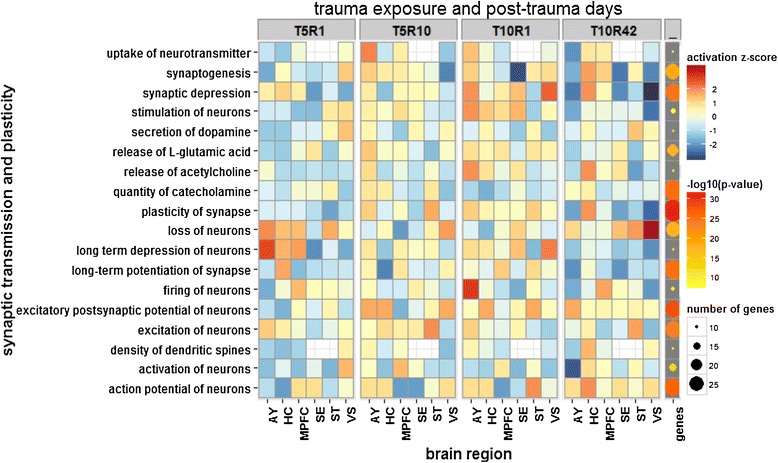
**Neuronal signaling, synaptic plasticity and neurogenesis associated with DEGs of brain regions at four different time points.**

The activation z-score for each enriched process at every time point showed increased activation of many of the anxiety-related behavioral responses at one-day post Agg-E (particularly in AY at T10R1) and a slight attenuation (but still an activated pattern) at longer rest periods (Figure 6). A similar approach for visualizing the signaling pathways related to synaptic plasticity and neurogenesis showed largely inhibited patterns (Figure 7).

Whereas we observed increased predicted activation of pathways implicated in behavioral responses, there was, in contrast, a decreased predicted activation in a broad spectrum of pathways pertaining to synaptic plasticity and neurogenesis (Additional file 1: Figure S7). There were, however, a few important exceptions such as loss of neurons, long-term synaptic depression (activated in AY, HC & MPFC) at T5R1, and activation of many of these signaling functions in AY at T10R1, and in HC (and to some extent in MPFC) at T10R42 (Additional file 1: Figure S7). This may be related to many of the elevated neuronal activities of AY since it is the center of fear and anxiety responses at one-day post Agg-E. On the other hand, after the longer rest period (T10R42), neuronal signaling may be more active in the HC due to long-term memory consolidation and related processes and in the MPFC since it is involved in regulating emotional responses.

Our assays and analyses showed that many genes related to PTSD-associated behaviors such as startle response (Figure 6 and Additional file 1: Figure S7), rearing, cognitive impairment, hyperactivity, anxiety, addiction, avoidance and fear conditioning (Additional file 1: Figure S7), had differential activation across tissues and time points, the larger activation generally being at one-day post-10 days of Agg-E (T10R1) sessions (Figure 6 and Additional file 1: Figure S7). Synaptic transmissions, including secretion and concentration of neurotransmitters, long-term potentiation, and synaptogenesis (Figure 7) were largely inhibited at one-day post-10 days of Agg-E (T10R1) and 10-days post-5 days of Agg-E (T5R10). We observed that neurotransmission and synaptic plasticity tend to be inhibited across tissues and time points compared to behavioral responses and neurological disorders, indicating impaired cognition and mood disorders (Figure 6), which are important facets of anxiety disorders.

### Networks of transcripts important in behavioral stress responses, synaptic transmission and circadian rhythm

Modular networks of DEGs from different brain regions have been significantly associated with associative learning, long-term memory, and regulation of serotonergic and dopaminergic synaptic transmissions, cognition, neurogenesis, limbic system development, startle and fear responses, and circadian rhythm (Figure 2). These pathways and biological processes are important molecular signaling implicated in traumatic responses leading to anxiety-related disorders.

### Comparative transcriptome changes among brain regions important in stress response circuitry

Transcriptome alterations in AY and HC were compared for overlapping and differential biological processes and pathways. These include a number of transcripts reported to be involved in startle and fear responses, social withdrawal, long-term memory, circadian rhythm, corticotropin releasing hormone receptor signaling, associative learning, aggressive and territorial behavior, and fear memory extinction (Figures 3 and 4 and Additional file 1: Figure S3). Red nodes show activation and blue nodes show inhibition of these particular behaviors. Many showed opposite regulation in AY compared to their expression in HC for the one-day post 10-days Agg-E session (T10R1) (Figures 3 and 4 and Additional file 1: Figure S3). These data suggest that there are opposing forces acting in AY compared to those molecular events in HC under severe stress or traumatic conditions possibly with incipient stress disorders.

### Common and specific transcripts for 5- and 10-day aggressor exposure sessions with different post Agg-E tissue collection rest (delay) days

Among DEGs from brain regions at different time points, 2,470 were associated with behavioral stress responses, synaptic plasticity and transmissions, circadian rhythm, obesity and diabetes, and inflammatory response. Of the 2,470 DEGs, 62 were specific to T10R1, 106 to T5R1, 103 to T5R10, 401 to T10R42, and 306 were common among all time points. The 306 common DEGs were significantly (Bonferroni, *q* < 0.05) associated with axon guidance, neurotransmitter secretion, opioid signaling, G-protein mediated events, downstream TCR-signaling, regulation of synaptic plasticity, associative learning, synapse assembly, CNS neuronal axonogenesis, long-term potentiation, long-term depression, activation of NMDA receptor and synaptic vesicles exocytosis (Additional file 1: Figure S8). The T5R1-specific 106 DEGs were associated with positive regulation of chronic inflammatory response, associative learning, dendritic spine development, negative regulation of axon extension, and the regulation of production of interleukins 10 and 12. The103 DEGs specific to T5R10 were associated with regulation of the perception of pain, response to carbohydrate stimuli, and organic acid transport. The T10R1-specific 62 DEGs were associated with positive regulation of circadian sleep/wake cycle, regulation of nerve growth factor receptor signaling pathways, negative regulation of cardiac muscle cell apoptosis, and cytotoxic activity; and the DEGs specific to T10R42 were associated with inflammatory and adaptive immune responses. It appeared that some of the biological processes and pathways relevant to traumatic stress responses started occurring early (during T5R1). On the other hand, most of the DEGs found only after the longer rest periods were associated with pain perception (T5R10) and the immune response (T10R42).

### Activation patterns of behavioral response and comorbid conditions

Traumatic stress disorder-related behaviors such as fear conditioning, anxiety-like behavior and startle responses showed differential activation patterns across the brain regions of AY, HC and MPFC at different time points (Figure 6 and Additional file 1: Figure S7). Fear conditioning-related genes were activated in the HC and MPFC (except T5R10) throughout the experimental time courses, but in the AY, this was activated at the early defeat period and attenuated at longer defeat periods (Figure 6). Early induction of transcripts implicated in fear conditioning suggested activation of this behavioral response early, consistent with the observation that AY responds first to traumatic stress. In the case of anxiety-like behaviors, transcripts associated with this process were induced in MPFC after the longer rest periods (T5R10 and T10R42), which is an indication of a delay in MPFC responses to anxiety-like disorders compared to AY (Figure 5). On the other hand, transcripts associated with anxiety show that AY responds at early periods and this response was greatly diminished with longer rest period after the traumatic event (Additional file 1: Figure S4). Although signs of inhibition of anxiety-like behavior at longer rest periods in AY indicated a sense of recovery, its activation in HC and MPFC in particular (Figure 5) suggested that it may precipitate in the forms of mood disorder and impaired cognition.

Comorbid conditions of anxiety disorders such as inflammation, obesity, diabetes and cardiac infarction were also among significantly enriched processes and pathways (Table 2). These observations suggest the pervasive and debilitating effect of resident-intruder social stress on the health of the Agg-E mice.

**Table 2:** **Differentially regulated genes (DEGs) significantly associated with different behavioral responses, neuronal signaling processes and comorbid conditions**

	***q*** **-Value**	**Associated DEGs**
Behavioral response		
anxiety	4.37E-17	APP,BDNF,CCK,CNR1,CREB1,CRH,CRHR1,DRD2, FKBP5,FOS,MAPK1,NPY,NTRK2
anxiety-like behavior	3.10E-10	APP,BDNF,CCK,CRH,CRHR1,MAPK1
cognition	9.54E-24	AKT1,APP,BDNF,CNR1,CREB1,CRH,CRHR1, DRD2,EP300,ERK1/2,FOS,GNAI1,HDAC3,KL,MAPK1,MAPK3,NGF,NPY,NR3C1,NTRK1,NTRK2,PI3K (complex),THRB
cognitive impairment	2.94E-10	APP,BDNF,CNR1,CRH,DRD2,LEP,NGF,NPY
conditioning	4.06E-12	APP,BDNF,CNR1,CREB1,CRHR1,DRD2,ERK1/2, GNAI1,HDAC2,MAPK1,NPY
depressive disorder	7.59E-10	APP,BDNF,CCKBR,CREB1,CRH,DRD2,FKBP5, NR3C1,NTRK1,THRB
emotional behavior	3.83E-15	APP,BDNF,CCK,CNR1,CREB1,CRH,CRHR1,DRD2, MAPK1,MAPK3,NGF,NPY,NTRK2
learning	1.25E-21	AKT1,APP,BDNF,CNR1,CREB1,CRH,CRHR1,DRD2, EP300,ERK1/2,FOS,GNAI1,HDAC3,HOMER1,MAPK1,MAPK3,NGF,NPY,NR3C1,NTRK1,NTRK2, PI3K (complex),THRB
locomotion	6.56E-11	APP,BDNF,CCKBR,CNR1,CRH,CRHR1,DRD2,LEP, NGF,NR3C1,STAT3
long-term memory	1.37E-15	APP,BDNF,CREB1,CRH,DRD2,ERK1/2,GNAI1, HDAC3,MAPK1,NTRK2
memory	5.63E-20	AKT1,APP,BDNF,CNR1,CREB1,CRH,CRHR1,DRD2, EP300,ERK1/2,GNAI1,HDAC3, HOMER1,MAPK1,NGF,NPY,NR3C1,NTRK1,NTRK2, PI3K (complex)
Mood Disorders	2.80E-10	AKT1,APP,BDNF,CCKBR,CREB1,CRH,DRD2, FKBP5,NR3C1,NTRK1,PLCG1,THRB
neurological signs	2.63E-11	AKT1,APP,BDNF,CCKBR,CNR1,CREB1, CTNNB1, DRD2,FOS,LEP,NGF,NPY,NTRK2,PRL
neuromuscular disease	1.45E-10	AKT1,APP,BDNF,CCKBR,CNR1,CREB1, CTNNB1,DRD2,FOS,LEP,NGF,NPY,NR3C1,NTRK2, PRL
object recognition memory	1.69E-09	APP,CNR1,EP300,HDAC3,NGF
post-traumatic stress disorder	5.32E-14	ADRA1A,ADRA1B,ADRA2A,ADRA2B,ADRA2C, ADRB1,CNR1,CNR2,DRD1,DRD2,DRD3,DRD4,DRD5,HTR1A,NR3C1,SLC6A4
recognition memory	2.36E-13	APP,CNR1,EP300,GNAI1,HDAC3,MAPK1,NGF, NR3C1
spatial learning	3.72E-10	APP,BDNF,CREB1,CRHR1,DRD2,FOS,NGF,NTRK2
spatial memory	2.95E-11	AKT1,APP,BDNF,CNR1,CREB1,DRD2,NGF,NPY, NR3C1,PI3K (complex)
Neuronal signaling and neurogenesis		
activation of neurons	1.22E-10	APP,BDNF,CCK,CNR1,CRH,FOS,LEP,NGF,PI3K (complex)
atrophy of neurons	4.63E-10	APP,BDNF,NGF,NTRK1,NTRK2
axonogenesis	1.84E-11	AKT1,APP,BDNF,CCK,CREB1,DRD2,FOS,NGF,NTRK1,NTRK2
cell death of hippocampal neurons	1.51E-09	AKT1,APP,BDNF,CRHR1,DRD2,LEP,NGF, NTRK2
concentration of corticosterone	1.28E-13	APP,CNR1,CRH,CRHR1,DRD2,LEP,NPY,NR3C1, NTRK2,STAT3
concentration of cyclic AMP	3.58E-09	ADCY,APP,BDNF,CCK,CNR1,CRH,DRD2,LEP,NGF, PRL
degeneration of neurons	2.07E-09	AKT1,APP,BDNF,CNR1,CREB1,EP300,NGF,NTRK2, STAT3
dendritic growth/branching	6.77E-11	AKT1,APP,BDNF,CNR1,CRH,CRHR1,DRD2,NGF, NTRK2
depolarization of cells	1.34E-10	APP,BDNF,CCK,CRH,FOS,LEP,NPY
disorder of basal ganglia	2.28E-10	AKT1,APP,BDNF,CCKBR,CNR1,CREB1, CTNNB1,DRD2,FOS,LEP,NGF,NPY,NTRK2,PRL
excitation of neurons	4.23E-12	APP,BDNF,CCK,CRH,FOS,NGF,NPY,NTRK2
length of dendrites	1.78E-12	APP,BDNF,CRH,CRHR1,NGF,NTRK2
long-term potentiation	4.96E-15	APP,BDNF,CNR1,CREB1,CRHR1,DRD2,ERK1/2, GNAI1,HDAC2,MAPK1,MAPK3,NGF,NPY,NTRK2, PI3K (complex)
long-term potentiation of synapse	1.44E-12	APP,BDNF,CNR1,CREB1,DRD2,GNAI1,HDAC2, MAPK3,NTRK2
loss of dorsal root ganglion cells	4.01E-10	BDNF,NGF,NTRK1,NTRK2
mobilization of Ca2+	3.95E-10	BDNF,CCK,DRD2,GNAI1,GNB2, inositol triphosphate,NGF,NPY,PI3K (complex), PLCG1,RXFP3,SRC
neuronal cell death	1.68E-15	AKT1,APP,BDNF,CNR1,CREB1,CRHR1,CTNNB1,DRD2,EP300,ERK1/2,FOS,HDAC3,LEP,MAPK1,NGF,NR3C1,NTRK1,NTRK2,PI3K (complex),SRC,STAT3
neuroprotection	4.24E-09	AKT1,APP,BDNF,CNR1,CRH,CRHR1,LEP,NGF,PI3K (complex)
neurotransmission	3.60E-17	APP,BDNF,CCK,CCKBR,CNR1,CRH,CRHR1, CTNNB1,DRD2,ERK1/2,GNAI1,HDAC2,LEP,NGF, NPY,NR3C1,SRC
proliferation of neuronal cells	2.13E-09	BDNF,CNR1,CTNNB1,DRD2,ERK1/2,LEP,NGF, NTRK2,PI3K (complex),STAT3
quantity of ACTH in blood	4.32E-09	CNR1,DRD2,NR3C1,NTRK2
quantity of neurons	4.71E-12	APP,BDNF,CREB1,DRD2,ERK1/2,LEP,NGF,NPY, NTRK1,NTRK2,PRL,THRB
quantity of pituitary cells	2.10E-11	CRH,CTNNB1,DRD2,LEP,PRL,THRB
release of acetylcholine	3.84E-09	APP,BDNF,CNR1,CRH,DRD2,NGF
release of Ca2+	3.82E-09	AKT1,APP,BDNF,CCK,DRD2, inositol triphosphate,LEP,NGF,PI3K (complex),PLCG1,SRC
release of catecholamine	3.32E-14	APP,BDNF,CNR1,CRHR1,DRD2,LEP,NGF,NPY, NTRK1,NTRK2,PRL,SRC
release of dopamine	1.84E-11	APP,BDNF,CNR1,CRHR1,DRD2,LEP,NGF,NTRK2, PRL
release of L-glutamic acid	1.12E-11	ADCY,APP,BDNF,CCK,CNR1,CRH,DRD2,LEP,NGF, NPY
secretion of catecholamine	2.06E-12	AKT1,APP,CNR1,CRH,CRHR1,DRD2,NPY,NTRK1
secretion of corticosterone	4.27E-12	BDNF,CRH,CRHR1,DRD2,LEP,NPY
secretion of neurotransmitter	3.44E-10	CCK,CNR1,CRH,DRD2,NGF,NPY,NTRK1,NTRK2
stimulation of neurons	4.28E-13	APP,BDNF,CCK,CRH,FOS,LEP,NGF,NPY,NTRK2
synaptogenesis	2.22E-10	APP,BDNF,CREB1,CTNNB1,HDAC2,NGF,NTRK1, NTRK2
synthesis of neurotransmitter	7.69E-10	AKT1,APP,BDNF,CRH,NGF,NTRK1
synthesis of steroid	3.71E-09	APP,BDNF,CREB1,CRH,CRHR1,LEP,MAPK1,NR3C1,PI3K (complex),PRL,PROX1
Comorbid conditions		
chronic inflammation	1.56E-11	ACE,ADORA2B,ADORA3,AGER,AOC3,C3,CCL5,CCR5,CD24A,CD28,CD47,CNR1,COX2,EDNRA,FABP4,FCER1A,FCER1G,FCGR1,FCGR3,GIF,HSPD1,IDO1,IL1B,ITGA2,JAK2,LBP,LTA,MIF,PDE5A,PIK3CG,PLA2G4A,PRKCA,PTGER3,PTGS2,STAT5A,STAT5B,TAC1,TGM2,TLR4,TNFRSF11A,TNFRSF1A,TNFSF11,H2-DMA,H2-DMB1,H2BFM,HADH,HNF1A,HPSE,HSPD1,HTT,ICA1,IFNGR2,IKBKG,IL12B,IL1R1,IL2,ILDR2,INPP5K,INPPL1,INSR,IRAK1,IRF1,IRS3,JAK2,KCNJ11,KHK,KRAS,LARS,LEP,LEPR,LEPROT,LIPE,LMAN1L,LPIN1,LTA, MAP3K7,MAPK8,MYD88,MYO5A,NCK1,NFKB1,NFKB2,NFKBIB,NFKB1,NFKB2,NFKBIB,RELA
myocardial infarction	6.49E-12	AKT1,AKT1,APP,CNR1,CREB1,CTNNB1,DRD2,EP300, ERK1/2,FOS,HDAC2,HDAC3,KL,LEP,MAPK1, MAPK3,NPY,NR3C1,STAT3,THRB,GPX3, SRC
hypertrophy of cardiomyocytes	6.16E-06	AKT1,EP300,HDAC2,LEP,MAPK1,PI3K (complex), STAT3
coronary artery disease	3.08E-04	APOE, CNR1,FOS,HDAC2,KL,NR3C1, LEP
metabolic disorders: types I and II diabetes	3.78E-15	AANAT,ABCC8,ACLY,ACSBG1,ACSBG2,ACSL4,ACSL6,ACVR1C,ADCY8,ADIPOR1,ADIPOR2,AGRP,AKT1,ANXA1,APAF1,APOE,ASIC2,ATM,BAD,BCAR1,CACNA1C,CAMP,CAPN8,CASP3,CBL,CCKAR,CD247,CDK2,CEBPB,CERK,CHUK,CITED1,CNR1,CNR2,CPE,CPLX3,GAD2,GCK,GH,GHRHR,GHRL,GHSR,GLP1R,GPR119,GRB2,GSK3B,GYS1,GZMB,H2DMA,H2DMB1,H2Q1,H2BFM,HADH,HNF1A,HPSE,HSPD1,HTT,ICA1,IFNGR2,IKBKG,ILDR2,INPP5K,INPPL1,INSR,IRAK1,IRS3,JAK2,KCNJ11,KHK,KRAS,LARS,LEP,LEPR,LEPROT,LIPE,LMAN1L,LPIN1,LTA,MAP2K2,MAP3K7,MAPK8,MC4R,MRAS,MTOR,MYO5A,NCK1,NGFR,NOS2,NPY,NRAS,NSMAF,OCRL,PAK1,SGK1,SH2B2,SIK2,SLC2A4,SLC2A8,SLC30A8,SMAD2,SOCS1,SORBS1,SORT1,SOS1,SOS2,SRC,STAT1,STAT3,STX1A,STXBP4,SYNJ2,SYP,TLN1,TLN2,TRADD,TRAF6,TRIB3,TRIP10,TSC1,TSC2,VAMP2,VDR,WDTC1,ZFP106
weight gain and obesity	4.33E-10	APP,BDNF,CNR1,CRH,DRD2,FOS,LEP,NPY,NTRK2, STAT3,CRK,CYB5R4,CYP27B1,DGAT1,DOK4,EGR1,EIF2AK3,EIF4EBP2,ENPP7,FADD,FAM3B,FAS,FASL,FCER1G,FFAR1,FKBP1B,FOXC2,FOXO4,FRS3,FYN,GAB1,PCLO,PCSK9,PDE3B,PDIA3,PDK1,PDPK1,PDX1,PHIP,PIAS1,PIK3C2G,PIK3CG,PIK3R5,PKLR,PKM,PLCB2,PLCZ1,PPARG,PPP1R3D,PRF1,PRKAR2B,PRKCD,PTEN,PTPN2,PTPRA,RAF1,RAPGEF4,RBP4,RELA,RETN,RHOQ,RIMS2,RIPK1,RPE65,RPS6KB2,RPTOR,RRAS2,SCAP,SCNN1G,

### Transcript – brain-region interactome across time points

**Figure 8:**
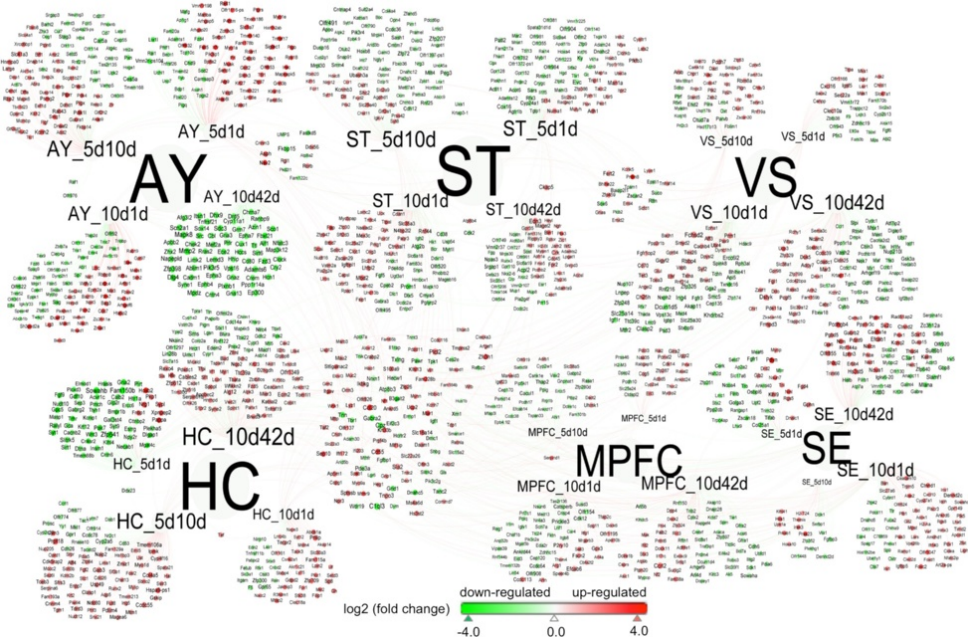
**Transcripts – brain-regions interactome for transcripts that passed p-value less than 0.01 and fold change greater than 2.0 in Agg-E groups compared to control groups (across time points).**

We constructed transcript – brain-region interactome using transcripts that passed p-value less than 0.01 and more than 2 fold across brain regions and time points (Figure 8). Persistent with what we observed with whole set of DEGs, the expression patterns of AY and HC were reversed for the early (5d1d) and for the late (10d42d) time points for transcripts filtered with stricter cut offs. This corroborates our suggested activation and inhibition of behavioral responses across time points among AY, HC and MPFC. Transcripts at early time point (5d1d) from AY were largely induced whereas they were suppressed in HC (Figure 8, Additional file 1: Figure S9). On the other hand, transcripts at later time point (10d42h) showed reversed expression pattern between AY and HC (Figure 8, Additional file 1: Figure S12), which is again consistent with our expression based prediction analyses. At the transcript level, there were not many shared DEGs among combinations of brain-regions and time points. But there were more overlaps at the pathway and process levels (Additional file 1: Figures S9-S18).

**Figure 9:**
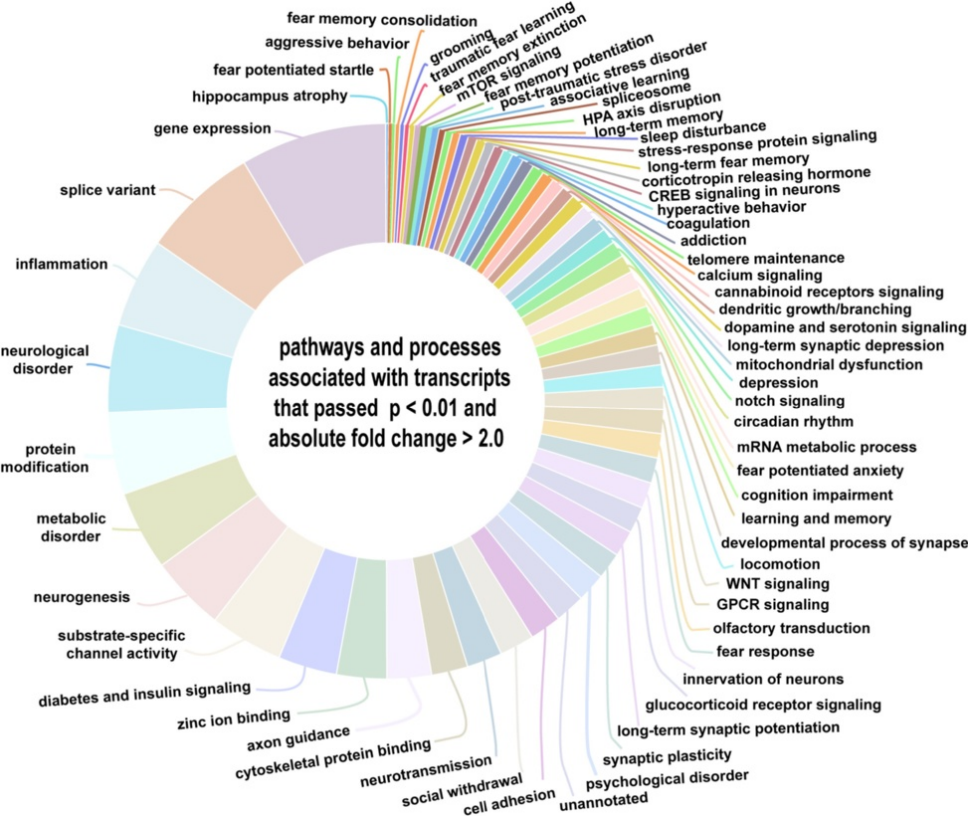
**Pathways and biological processes significantly associated with transcripts (with p-value < 0.01, and fold change > 2.0) shown in Figure** **8** **.** Sizes of the hollow-pie sectors were proportional to the number of transcripts associated with that particular pathway or process. Gene expression related processes such as activities of zinc finger protein transcription factors, and splice variant processing were associated with larger numbers of transcripts (and many of these were also shared with pathways related to trauma/fear related behavioral responses).

A large portions of these sets of transcripts were associated with splice variant processing and activities of zinc finger transcription factors (Figure 9 and Additional file 3: Table S2). Many of these transcripts were also shared also with those transcripts suggested to be involved in traumatic disorders and comorbid conditions (Figure 9 and Additional file 3: Table S2).

### Validation of gene expression profiles

Transcripts implicated in anxiety and fear responses in PTSD patients and animal models were assayed in each brain region (AY, HC, MPFC, SE, ST and VS) at the T10R1 time point using the QuantiGene Plex 2.0 Reagent System (Multiplex) to corroborate our microarray results. Many of the samples assayed by the QuantiGene Multiplex showed comparative direction of expression with that of microarray data (Additional file 1: Figures S19.a and S20). Transcripts critical in nerve growth factor and synaptic plasticity such as FK506 binding protein 5 (Fkbp5), brain-derived neurotrophic factor (Bdnf), thyroid hormone receptor beta (Thrb) and prostaglandin-endoperoxide synthase 2 (Ptgs2) were largely suppressed in both microarray and QuantiGene assays. Transcripts of the neuronal signaling molecules (substrate specific channels) such s Slc1a2 was up-regulated and Il1rap was down-regulated in all regions except in AY, while Rxfp3 and Prox1 were up-regulated in AY, HC and MPFC regions (Additional file 1: Figures S19.a and S20).

Correlation analysis for each transcript between the bead and array data showed directional correlation for the transcripts: Fkbp5, Thrb, Il1rap, Bdnf, Drd2, Slc1a2, Coch, Gng4, Rxfp3 and Slc1a2; while Gpx3, Npy, Prox1 and Ptgs2 showed lower directional correlation between the two platforms (Additional file 1: Figures S19 and S20). Rxfp3, Gng4 and Slc1a2 showed overall opposite correlation compared to the rest of the transcripts as shown by the correlation matrix of all the assayed transcripts (Additional file 1: Figure S19.b). These transcripts were mostly up-regulated in both platforms (red ellipses) where as the other transcripts showed similar correlation and were largely down-regulated in the two platforms (blue ellipses) (Additional file 1: Figure S19.b). This also corroborated the notion that neurogenesis related brain factors were suppressed whereas some of transcripts implicated substrate specific channels and neuronal signaling were induced at the molecular level.

## Discussion

Cellular and molecular changes in the activity and functional connectivities among the amygdala (AY), hippocampus (HC), medial prefrontal cortex (MPFC) play primary roles in fear learning and memory, and thus, may contribute to the behavioral manifestations of anxiety disorders (Additional file 1: Figure S1).

Identification of the molecular basis of learning and memory mechanisms across stress-induced anxiety disorders is critical for devising means of mitigating the severity of traumatic disorders. This is because both memory retrieval (involved in symptom expression, reconsolidation, and maintenance of these disorders), and memory extinction (believed to be the mechanism of behavioral exposure therapy of anxiety disorders) are dependent on transcriptional changes of the underlying molecules governing synaptic plasticity and inter-neuronal signaling.

Transcripts significantly associated with synaptic transmission and plasticity, long-term depression and potentiation, axonogenesis, and the synthesis and release of neurotransmitters such as glutamate, acetylcholine, dopamine and serotonin were differentially regulated across time points and brain regions due to the effects of social stress trauma on Agg-E mice. These molecular changes are corroborated by changes in other groups of transcripts associated with behavioral expression of anxiety disorders such as startle response, anxiety, hyperactivity, fear conditioning, emotional behavior, cognitive impairment, and addiction and alcohol abuse-associated behaviors. Molecular indicators of addiction and alcohol abuse showed increased activation z-scores (Additional file 1: Figure S7A), which correlates with the observations of others that traumatic events sufficient to produce long-lasting enhancement of fear learning also increase voluntary ethanol consumption [36].

DEGs involved in synaptic transmission in relation to long-term synaptic potentiation, long-term synaptic depression, and synaptic plasticity are the basis for learning and memory consolidation. These pathways and functional enrichment findings may indicate the acquisition, consolidation and maintenance of traumatic memories to form anxiety disorders via fear learning and fear memory circuitry, as well as deficiencies in extinction and re-extinction mechanisms along the different time course trajectories in different brain regions. For example, the AY had higher activation z-scores of anxiety-related behaviors at earlier time points compared to the MPFC, which corresponded with the earlier response in the amygdala. This supports the belief that neural circuits underlying PTSD are characterized by exaggerated AY activation and decreased MPFC activation, leading to the elevated anxiety state and concomitant inadequate emotional regulation [37]. Re-experiencing the traumatic event in the form of recurrent distressing images and recollections, including the intrusive traumatic memory, is unique to PTSD among anxiety disorders [17,38], and is probably due to diversion of focus from the current stimuli (caused by impaired cognition due to slow acting MPFC) to the intrusive traumatic event (interference effect of the faster paced AY activities).

In addition, DEGs reported to be involved in the fear-potentiated startle response, fear extinction resistance, associative fear learning, and cue conditioning were indicative of the formation of traumatic memories and a vulnerability to developing fear-related anxiety disorders. For example, the transcripts FKBP5 and BDNF were suppressed across the six brain regions at T10R1 (Additional file 1: Figures S19.a and S20). FKBP5 is involved in the behavioral and neuroendocrine effects of chronic social defeat stress. Suppression of FKBP5 expression in HC leads to persistent traumatic disorder and confers fear extinction resistance [22,23]. BDNF is a crucial regulator of neuroplasticity, which underlies learning and memory processes in different brain areas. Chronic suppression of BDNF expression in the HC, AY and the prefrontal cortex leads to a deficit in the acquisition of extinction memory, and increased BDNF expression supported amelioration of hyperarousal in a mouse model of PTSD [39]. BDNF facilitates extinction learning, and decreased mRNA expression of BDNF within the MPFC leads to resistance to fear extinction. Activation of BDNF signaling by the TrkB agonist 7, 8-dihydroxyflavone blocks the return of fear after extinction training [40,41].

The exact social stress process that we used here was also shown to result in decreased BDNF in the HC, which led to suboptimal binding of BDNF to tyrosine receptor kinase B (TrkB), resulting in curtailing downstream intracellular signaling pathways, including mitogen-activated protein kinase/extracellular signal-regulated protein kinase (MAPK/ERK), phospholipase Cg (PLCg), and phosphoinositide 3-kinase (PI3K) pathways. These pathways are important for neurogenesis and cognition [42,43]. Another important DEG along this pathway is the nerve growth factor, Ngf, that also shown to bind to tyrosine receptor kinase A (TrkA). In response to Ngf, the TrkA forms a complex with Shc, coupling TrkA to p21ras and Shc with Grb2, which is mediated by autophosphorylation of TrkA [44]. However, suppression of Ngf at earlier time points (T5R1 and T10R1) suggestive of curtailed kinase activities and complex formations, potentially leading to impaired synaptogenesis.

Potential contribution to the etiology of PTSD may be due to sensitization of glucocorticoid receptor (GR) signaling and dysregulation of GR modulators [45] such as FKBP5 and Crf1 receptors. Furthermore, Akt, Nfkb and MAP kinases, which are G protein-coupled receptor (GPCR) pathway molecules, can promote or prevent sustained high anxiety- and depressive-like behaviors following severe stress. Agonist-induced activation of the corticotropin releasing factor (Crf1 receptor) is crucial for survival in the context of serious danger or trauma, but persistent Crf1 receptor hyper-signaling when a threatening or traumatic situation is no longer present is maladaptive. Also, suppressed expression of Grk3, which phosphorylates the Crf1 receptor protein indicates there was suboptimal binding of Crhr1 with beta-arrestin2, leading to inhibited termination of Grk3-coupled Crf1 receptor signaling by homologous desensitization. This deficiency in Grk3-beta-arrestin2 complex formation is suggested to contribute to PTSD and co-morbid post-traumatic depression [46].

FKBP5 is also shown to mitigate HPA axis dysfunction [47]. Molecular changes involved in modulation of the HPA axis are associated with cue conditioning, calcium signaling, memory processes, and regulation of the stress responses. Transcripts of genes involved in HPA axis, such as those related to corticotrophin releasing factor (CRF) receptors pathways, cortisol (glucocorticoid) receptor signaling and oxytocin receptor (such as Nr3c1, Nr3c2, Crhr1, Crhr2, Oxtr) were also differentially regulated. Oxytocin (mammalian neuropeptide) modulates activation of fear extinction-based neural circuits and fear responses [48].

Release of GC, CRF and modulators are shown to affect emotional learning and memory in relation to etiologies of anxiety disorders. The pathogenesis of PTSD is attributed to over-consolidated traumatic memories that are mediated by endogenous stress hormones released during and after trauma. Downstream secondary messenger signaling pathways with a putative role in long-term potentiation (LTP), such as inositol 1,4,5-trisphosphate (IP3) and diacylglycerol (DAG) with the upstream enzyme inositol monophosphatase (Imp), favor fear learning and traumatic memory consolidation in traumatized and susceptible individuals via fear potentiated LTP, and the eventual development of PTSD [49]. Glucocorticoid increase after longer rest periods in Agg-E mice is probably an indicator of attenuated conditioned fear and is in agreement with the observation that stress-induced circulating cortisol was shown to reduce memory retrieval of conditioned fear in men [50].

Transcript levels of genes reported to be associated with cognition and object recognition (in particular, suppressed expression of BDNF, Gnai1, App, Ep300, Cnr1, Cnr2 and Ngf transcripts) at one-day post-10 days of Agg-E session (T10R1) indicated there was impaired cognition, and therefore, inhibition of novel object recognition. The memory modulating cannabinoid receptors (Cnr1 & Cnr2) and elevated levels of endocannabinoids (endogenous cannabinoids) in the basolateral AY and have a critical function in the extinction of aversive memories. Cnr1-deficient mice showed strongly impaired short-term and long-term extinction in auditory fear-conditioning tests, with unaffected memory acquisition and consolidation. Treatment of wild-type mice with the Cnr1 (CB1 receptor) antagonist SR141716A mimicked the phenotype of CB1-deficient mice, suggesting that CB1 is required at the moment of memory extinction. In the basolateral AY, endocannabinoids and CB1 were crucially involved in long-term depression of GABA (gamma-aminobutyric acid)-mediated inhibitory currents [51,52].

Alternatively, transcripts of the histone deacetylases (Hdac2 & Hdac3) showed increased expression, also indicating impaired object recognition. Impaired object recognition memory may underlie certain avoidant symptoms or negative cognitions in PTSD and be related to impaired behavioral flexibility [53]. Impaired novel object recognition parallels neurocognitive deficit (impaired memory and attention) in PTSD patients who show lower performance on memory tests specific to learning and executive functions [54].

Other important DEGs include cFos and its (promoter) histone deacetylase enzyme, Hdac2, GluN2B (Dusp1), and klotho (*Kl*). Hdac2 and cFos play a critical role in fear memory recall for reconsolidation and updating acquired memories in neuronal plasticity [38,39]. A similar social defeat model showed that presentation of an aggressor cue induced robust increases in cFos in AY, MPFC and *CA1* of HC both 1 and 7 days after Agg-E. The increase at 7 days is greater than that at one day, whereas avoidance behavior was great at one day post-Agg-E and had somewhat abated by 7 days post-Agg-E. This suggests that increased neuronal processing was related to the decrease in avoidance [55]. In the conditioned fear paradigm of mice, fear memory extinction is shown to be more effective in a one-day-old fear memory compared to a 30-day-old fear memory. Older fear memories are less labile due to hypoacetylated c-Fos promoter by Hdac2 in HC [56]. Among others, this may be one reason why we observed more DEGs after 42 days of rest (T10R42) compared to one-day post 10-day Agg-E sessions (T10R1), with related DEGs activated upon remote memory recall [39,40]. Elevated *K1* in mice has been shown to enhance long-term potentiation (synaptic plasticity), and enrich synaptic GluN2B (an NMDA receptor subunit) with key functions in learning and memory. Blockade of GluN2B abolished klotho-mediated effects. Suppressed expression of *K1* may indicate cognitive deficits in our model animals [57].

Overall, transcripts of DEGs in Agg-E groups of mice compared to controls in the different brain regions were reported to be associated with glucocorticoid negative feedback signaling, arousal to trauma cues (jumping), impaired aggressive behavior (avoidance of aggressor-cued partition), social withdrawal, impaired territorial behavior, long-term fear memory, grooming, decreased movement, anxiety, long-term synaptic depression and potentiation (long-term synaptic plasticity), inhibited dopaminergic signaling, HC atrophy, dendritic branching in AY, conditioned fear association, fear memory consolidation, retrieval and impaired contextualization, sensorimotor gating deficit, deficit of executive function, impaired object recognition, and circadian rhythm disruption (Table 2).

Significant association of DEGs with comorbid conditions, such as chronic inflammation, myocardial infarction, suppressed protective immunity and obesity/diabetes indicate the pervasive nature of Agg-E, leading to many systemic pathological consequences. Previous studies using this same mouse model simulating PTSD showed acute myocardial injury associated with the traumatic experience as a consequence of underlying biological injury processes, including inflammation [58]. Metabolomic, histopathology and liver transcriptomic analyses showed increased inflammatory response at one-day post-stress, persistent myocarditis and cardiac fibrosis, and hyperlipidemia in the liver, related to risks of heart disease and obesity [59]. Similar co-morbidities of PTSD observed in veterans and active duty personnel include inflammation, obesity, diabetes, and heart disease [3], as well as sleep disorders [60,61] and higher rates of pain, possibly due to chronic inflammation [62].

Altered trauma induced behavioral responses and associated comorbid pathways (discussed before and additional pathways) were also significantly associated with the more significant transcripts (with p < 0.01, and fold change > 2.0) (Figure 9 and Additional file 1: Figures S9-S18). Additional pathways related to splice variant processing, mitochondrial dysfunction, metabolic disorder, substrate specific channel activities, activities of zinc finger related transcription factors and cytoskeletal protein binding were significantly enriched (Figure 9). Association of many of the top transcripts with splice variant processing may hint to the basis of molecular mechanisms leading to either stress resilience or susceptibility among individuals. Transcripts associated with mitochondrial dysfunction along with those involved in metabolic disorder may contribute towards exaggerated fear in traumatized individuals [63].

## Conclusions

Pathways and functions presumably important for the etiology of PTSD-like disorders, including endocannabinoid signaling, HPA axis function, modulators and targets of cortisol, neuronal transmission, neurogenesis and fear memory extinction with regard to emotional learning and memory, and a number of anxiety-related behavioral responses were significantly associated with DEGs.

Activation z-scores of anxiety-related behavioral responses, fear acquisition and consolidation-related signaling pathways and processes reveal the distinct, and probably complementary roles of early time points versus later rest periods (of 5-day and 10-day Agg-E sessions) in the traumatic fear-learning trajectories. The molecular mediators of the earlier stress responses (first-wave responders) seem to enhance fear maintenance (in AY), whereas the second-wave of transcripts seem to either consolidate fear memory (in HC) or attenuate fear learning (in MPFC).

### Future directions and suggested works

Advanced experimental tools, such as inducible mutations in mice, virus-mediated gene transfer, and optogenetics provide the means to directly delineate the role of specific proteins acting within specific cell types of anxiety-processing brain structures in mediating PTSD-like behavioral abnormalities in animal models.

There is evidence that females use different cognitive strategies, exhibit increased stress sensitivity, and show variations in reward-related behaviors throughout the estrus cycle that may render them more sensitive to the deleterious effects of stress. Future studies may examine the molecular and cellular underpinnings of anxiety-like behaviors in females. For example, depression is twice as likely to occur in women as in men [24], and this may be true for other anxiety disorders as well, but animal studies including ours have mostly been conducted in males.

Untreated traumatic fear memory in susceptible individuals precipitates in the form of anxiety disorders such as PTSD. One effective treatment of anxiety disorder is using exposure therapies, which are essentially traumatic fear memory extinction and are effective during the labile phase of fear memory consolidation. Since the window of fear memory consolidation is relatively short compared to the enduring flashback vulnerability, remote fear memories may not be persistently unlearned by reconsolidation-updating paradigms. Hence, there is a need to identify molecular mediators of stable fear memory in order to devise a mechanism of extinction of remote fear memory long after its consolidation window.

## Methods

### Exposure of subject mice to aggressor mice

Albino SJL male mice (The Jackson Laboratory, Bar Harbor, ME) were single-housed for one month and trained as aggressors by placing bulbectomized C57BL/6 male mice in SJL cages as intruders. [Note: the purpose of bulbectomized C57BL/6J mice (which don’t fight back) was only to train the SJL mice to be more aggressive]. Naïve C57BL/6J male mice single-housed in a separate room were used as experimental subjects (5 exposed and 5 control mice per time point were used). Each subject mouse was kept in a protective mesh box inside the cage of the aggressor SJL mouse for 6 hours/day for 5 (T5) or 10 days (T10), with direct exposure to the aggressor mouse for either 1 minute or 10 bites (whichever occurs first) for 1–3 random times during the 6-hour defeat session. Control (C-ctrl) mice were also housed without feed and water for the same period in a room separate from the aggressor mice.

Body weights and temperatures of the experimental mice were measured (using implanted electronic ID transponders) before and after the social defeat session of each day. Details are given in separate publications [29]. Territorial urine markings were measured by placing blotting papers (0.8 mm thick) under the cages of Agg-E and C-ctrl mice. Areas were compared as described in [29].

### Assays for corticosterone levels

One hour after the partition test, mice were euthanized by cervical dislocation and blood samples were immediately drawn by cardiac puncture and collected in nuclease-free tubes containing 50 μl of buffered sodium citrate (0.105 M ~ 3.2%). Whole blood samples were then centrifuged and plasma was separated and stored at −80°C until the corticosterone assay was performed. Corticosterone level was measured using an ELISA kit following the manufacturer’s instructions (Kamiya Biomedical Company, Seattle, WA).

### Brain dissection and collection of brain regions

Brain regions from subject and control C57BL/6 mice were collected after one (R1) or 42 days (R42) of post 10-day (T10) aggressor exposure, and one (R1) or 10 days (R10) after the 5-day (T5) aggressor exposure. Brains were carefully removed from the skulls, and the left or right hemi-brain from each traumatized or control mouse was dissected into different anatomical and functional regions: amygdala (AY), hippocampus (HC), medial prefrontal cortex (MPFC), ventral striatum [including nucleus accumbens] (VS), septal region (SE) and corpus striatum (ST). Collected tissues were immediately frozen in liquid nitrogen and stored at −80°C for total RNA isolation.

### RNA isolation

Total RNAs were isolated according to the Trizol® method (Invitrogen, Inc., Grand Island, NY) from homogenized brain regions. Isolated RNAs were stored at −80°C until microarray assays were conducted.

### Microarray hybridization

Microarray assays were performed using Agilent’s genome-wide mouse expression array (GE 4x44K v2 two color microarray) slides and kits (Agilent Technologies, Inc., Santa Clara, CA) following the manufacturer’s protocol. Hybridized microarray slides were scanned using Agilent Technologies Scanner G2505C US09493743.

### Feature extraction and normalization

Images of scanned microarray slides were feature-extracted and normalized using Agilent’s feature extraction software, version 10.7 or later, in the default setup (Agilent Technologies, Inc.).

### Microarray data analysis

Normalized data were filtered on flags to exclude probes, which have missing values in more than one sample, and these were tissue-wise quantile normalized using the Limma Package of R program (www.r-project.org). Normalized data were analyzed using Agilent’s Genespring GX v12.0, the Limma package of R, and Qlucore Omics Explorer 2.3 (Qlucore AB, Lund, Sweden). Each brain region at each time point (T5R1, T5R10, T10R1, and T10R42) was compared for aggressor exposure effects: Aggressor-Exposed (Agg-E) vs. Control (C-ctrl). DEGs in each tissue and time point were identified using a Moderated T-test at *p* < 0.05 of the Limma Package of R.

Time effects were also compared using TimeClust [64] across different combinations of aggressor exposure sessions and rest periods (T5R1, T5R10, T10R1, and T10R42).

### Behavioral association of DEGs with respect to functional and pathway enrichments

To assess behavioral association of DEGs, we followed two complementary approaches:

i. We started with DEGs across different brain regions and identified significantly enriched signaling pathways and biological processes.
ii. We used frequency calculation to determine psychopatholy-related pathways and biological processes cited in the scientific literature in relation to social stress, trauma, PTSD, anxiety, and depression. We then identified (from databases and literature) transcripts and proteins directly associated with the most frequently cited terms. Literature- and database-mined genes and proteins were intersected with DEGs from our study (brain regions), and those in common were used to determine probable downstream biological processes, as well as networks and pathways and their significant associations.

### Gene ontology and pathway enrichments, regulatory networks analysis, common pathways analysis, and visualization

The Hypergeometric Test (false discovery rate (FDR), *q* < 0.05) of Bingo 2.44 and ClueGO (Cytoscape 3.0.1 plug-ins; http://www.cytoscape.org/), Fisher’s Exact Test of Ingenuity® (Ingenuity, Inc., Redwood, CA), and Gene Set Enrichment Analysis (http://www.broadinstitute.org/gsea/index.jsp) were used for gene ontology and pathway enrichments. The Cytoscape and Ingenuity Pathway analytical software systems were also used for network analyses and visualizations. Common pathways were intersected and visualized using Cytoscape and graphically displayed using the R programming (www.cran-r.org) graphic system. Activation z-scores of behavioral responses and synaptic plasticity were used to identify induced and suppressed behaviors and synaptic activities across brain regions and time points.

Transcript – brain-region interactomes were constructed and visualized using Gephi.0.8.2 beta (www.gephi.org). Functions and pathways associated with the top transcripts (p-value < 0.01, and fold change > 2.0) were enriched using ClueGo, Bingo, IPA and David (using FDR correction of q < 0.05). Bar charts of the corresponding processes and pathways were constructed using the ggplot2 of R.

### Gene expression profiling using QuantiGene Plex 2.0 Reagent system

Target–specific RNA molecules (*Thrb*, NM_009380; *Ptgs2*, NM_011198; *Prox1*, NM_008937; *Drd2*, NM_010077; *Slc1a2*, NM_011393; *Rgs4*, NM_009062; *Hmga2*, NM_010441; *Npy*, NM_023456; *Rxfp3*, NM_178717; *Gng4*, NM_010317; *Bdnf*, NM_007540; *Coch*, NM_007728; *Il1rap*, NM_134103; *Gpx3*, NM_008161; *Fkbp5*, NM_010220) from different brain regions were analyzed by the QuantiGene Plex 2.0 Reagent system (Affymetrix, Fremont, CA). Oligonucleotide probe sets (capture, label, and blocker probes) for each gene were designed by the manufacturer. Briefly, RNAs from different brain regions were captured by fluorescent microspheres. After overnight hybridization at 54 ± 1°C, hybridizations with branched DNA (bDNA) pre-amplifier 2.0, bDNA amplifier 2.0, biotinylated label probe and, finally substrate were sequentially carried out according to the manufacturer’s instructions. Signals of cascade amplification were detected by Bio-Plex 100 x MAP technology and analyzed using Bio-Plex 6.0 software (Bio-Rad Laboratories, Hercules, CA). Mean fluorescence intensity (MFI) signals generated from each bead are proportional to the amount of each mRNA captured on the surface of each generated specific probe set [65].

The geometric means of four housekeeping genes (*Ppib*, NM_011149; *HPRT1*, NM_013556; *Ldha*, NM_010699; *Rplp0*, NM007475) were used for normalization of each sample. Fold-changes were the relative ratios between normalized values of treated groups and that of the control group. Correlations between QuantiGene and microarray data were calculated using GraphPad Prism® 5.04 (GraphPad Software, Inc., La Jolla, CA).

The *Pearson product moment (linear) correlation coefficient and correlation significance* for each transcript in the microarray and Quantigene bead platforms were calculated using base package of R. Correlation matrix was calculated and plotted using corrplot package of R.

### Data deposition

All datasets used in the study have been deposited in Gene Expression Omnibus of NCBI [GEO accession #: GSE45035] [http://www.ncbi.nlm.nih.gov/geo/query/acc.cgi?acc=GSE45035].

## Additional files

Additional file 1:
**Supplemental Figures S1-S20.**
Additional file 2: Table S1. Pathways and process associated with differentially regulated transcripts across brain regions and time points (Note: the p-value is the gene level; but the pathways and processes were corrected using FDR at q < 0.05). Additional file 3: Table S2. Pathways and process associated with differentially regulated transcripts that passed p < 0.01 and fold change > 2.0 (across brain regions and time points) (Note: the p-value is the gene level; but enrichmenst of the associated pathways and processes were corrected using FDR at q < 0.05).

## Abbreviations

PTSD: Post-traumatic stress disorder
Agg-E: Aggressor-exposed (social stressed)
C-ctrl: control
DEG: Differentially expressed gene
HC: Hippocampus
AY: Amygdala
MPFC: medial prefrontal cortex
VS: Ventral striatum, includes nucleus accumbens (NAc)
SE: Septal region
ST: Corpus striatum
T10 and T5: 10 days and 5 days of aggressor exposure sessions
R1, R10 and R42: One, 10 and 42 (post-Aggressor exposure) days, respectively
GPCR: G-protein-coupled receptors
FDR: False discovery rate
