# Supplementary material for: Brain transcriptome profiles in mouse model simulating features of post-traumatic stress disorder
Source: Mol Brain. 2015 Feb 28;8:14. doi: 10.1186/s13041-015-0104-3 (PMC4359441; doi:10.1186/s13041-015-0104-3)
Supplement: Additional file 1: — Supplemental Figures S1-S20. [file 13041_2015_104_MOESM1_ESM.pdf]

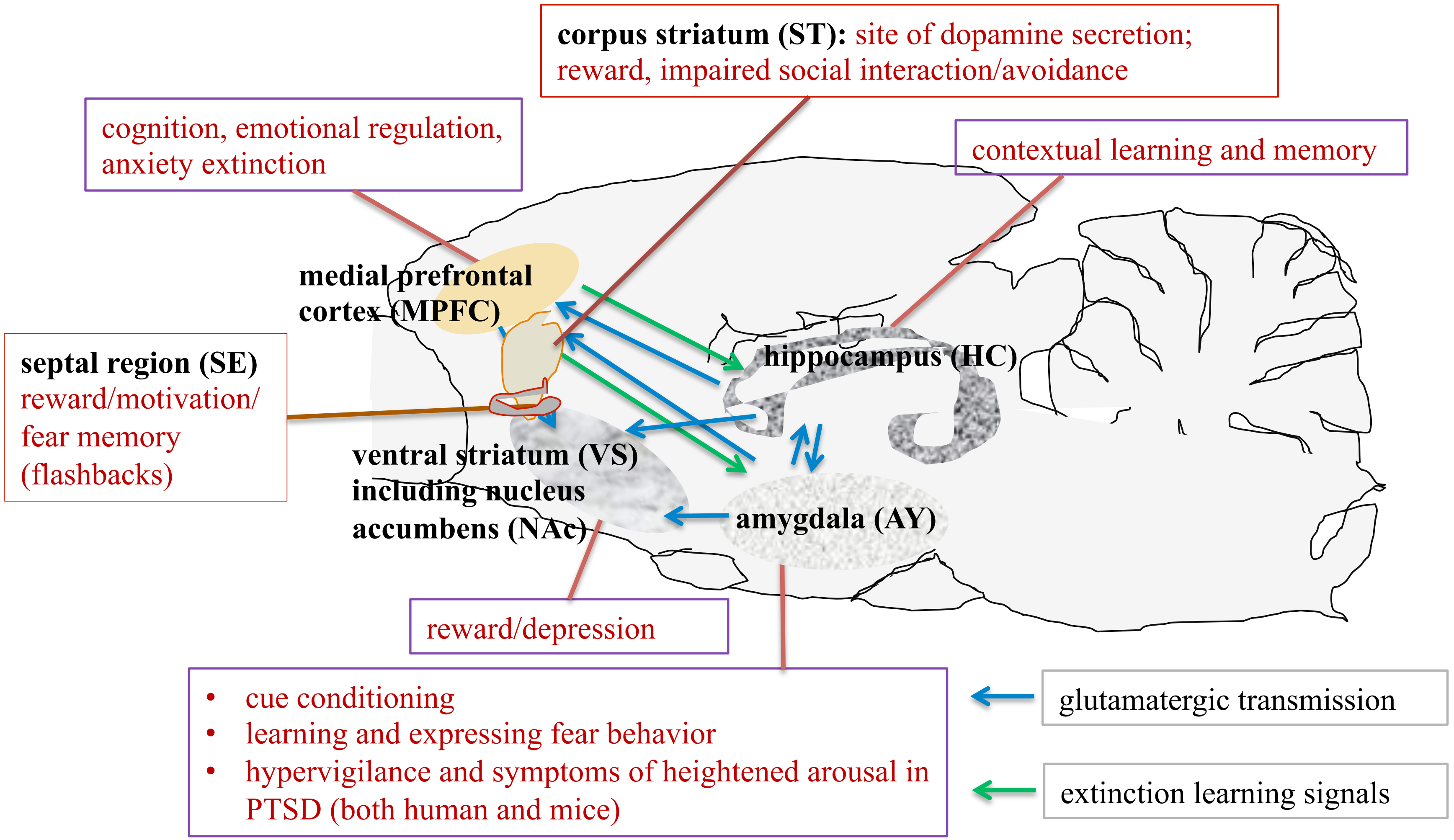

Figure S1: Mouse brain regions important for anxiety and mood disorder used for genome-wide transcriptome profiling. Molecular and cellular changes in the activity and functional connectivity of these regions are the basis for fear learning and memory, leading to susceptibility to traumatic disorders.

Profiles of genome-wide transcriptome changes in trauma-responsive brain regions were carried out at different time points. These assays were designed to assess how the time course dynamics of transcripts indicate molecular events associated with traumatic fear learning and memory along the traumatic induced anxiety disorder trajectories.

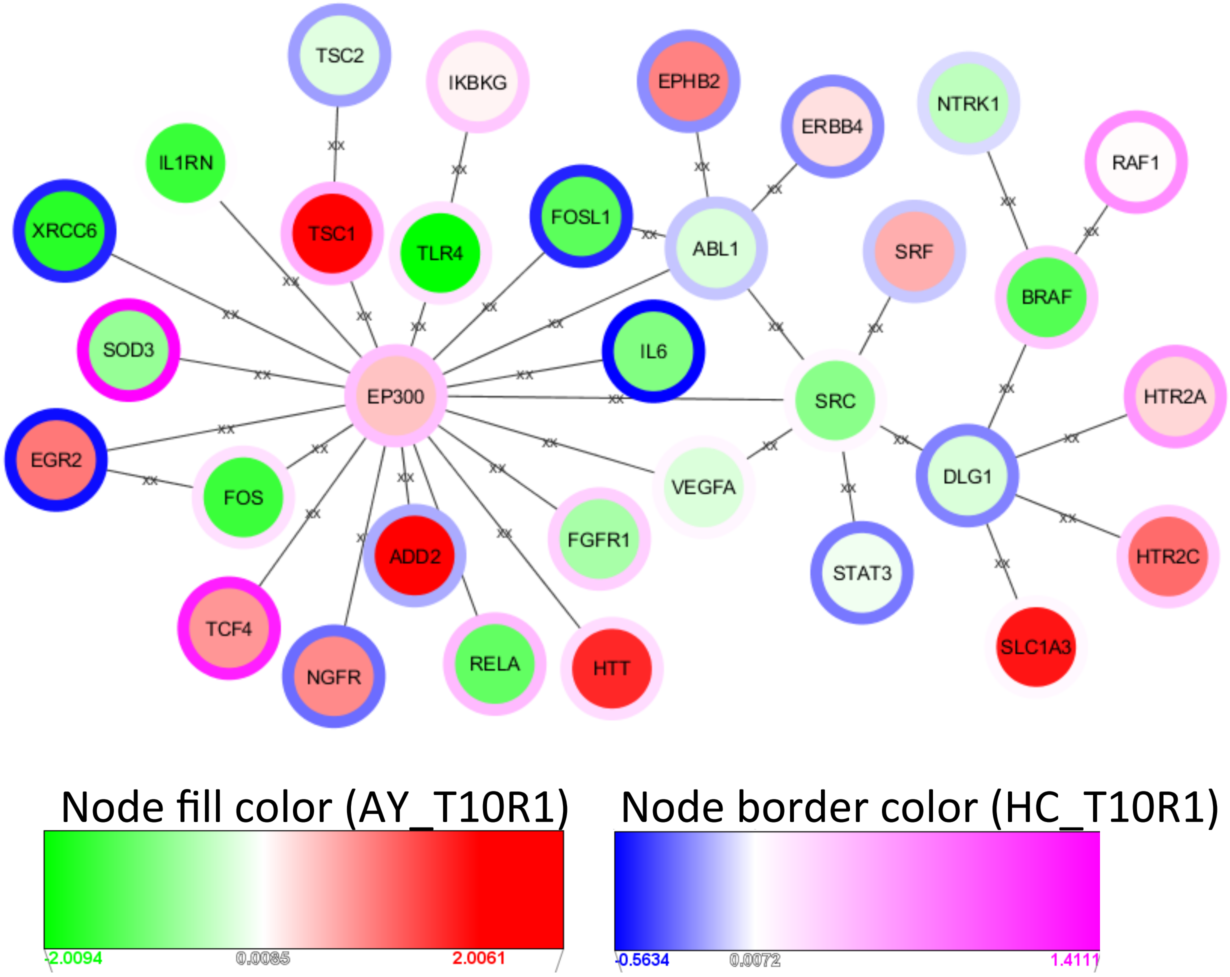

Up-regulated (in AY_T10R1)
-neurotransmitter binding (serotonin and l-glutamate)
-glucocorticoid receptor binding
-neurotrophin receptor activity and axonal guidance

Down-regulated (in AY_T10R1)
-inflammation
-superoxide dismutase
-growth factor receptors and metabolism

In HC_T10R1, some of the nodes follow the expression pattern of AY_T10R1 while others regulated in opposite direction.

Figure S2: Network of genes significant in AY_T10R1, and colored using their AY_T10R1 and HC_T10R1 expression levels

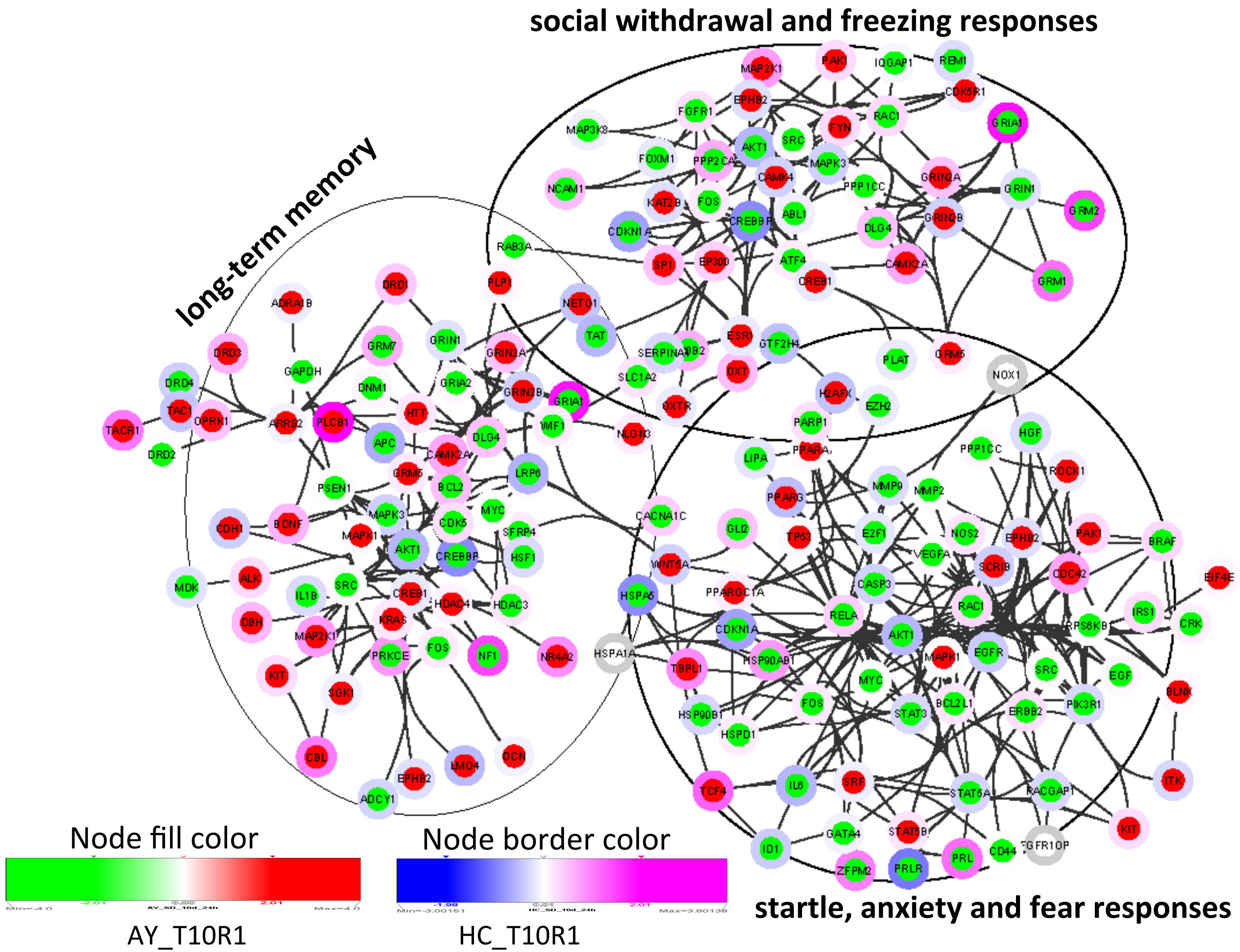

Figure S3 Networks of DEGs significantly associated with long-term fear memory, social withdrawal, startle and fear responses. Nodes are colored using AY_T10R1 and HC_T10R1.

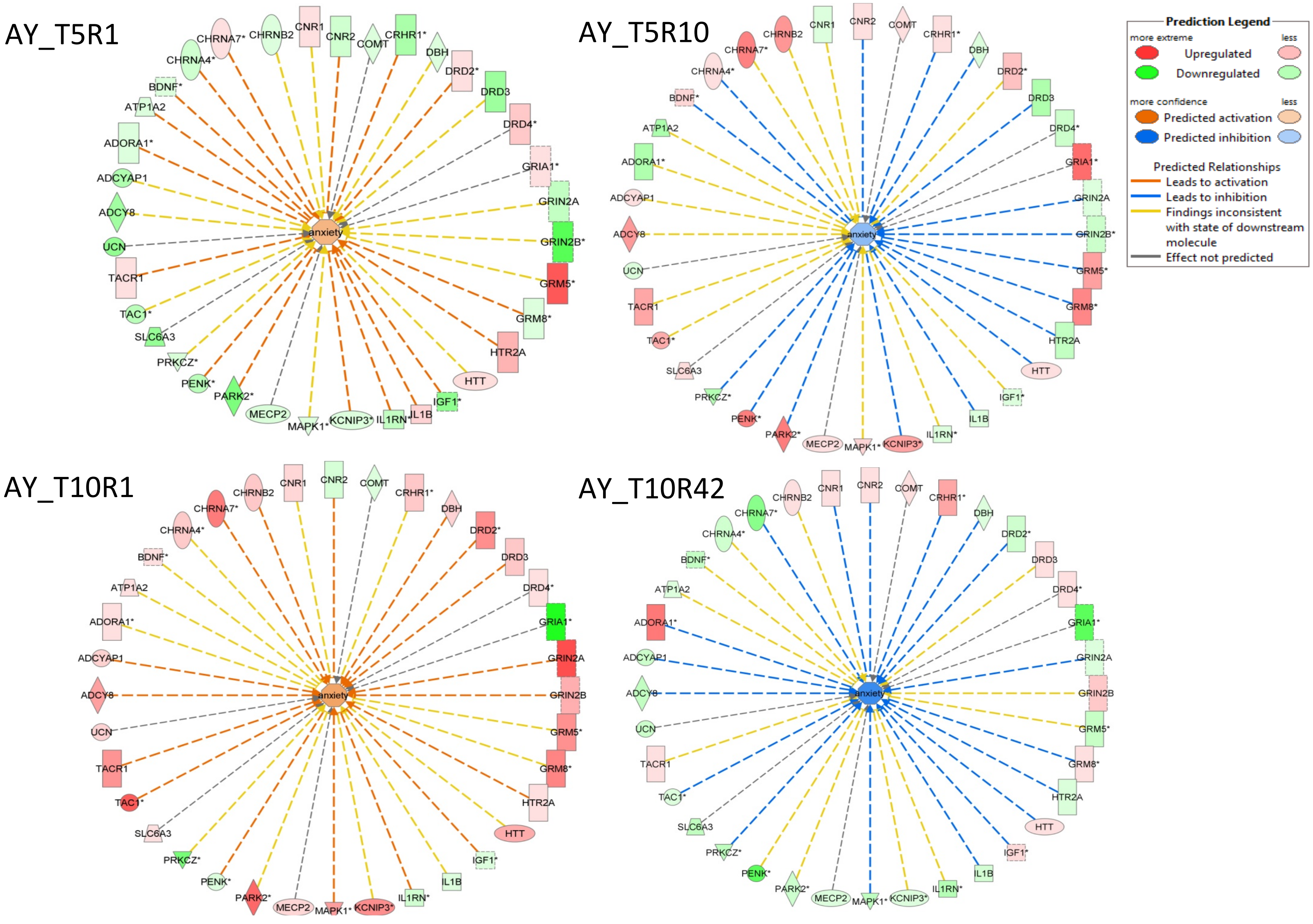

Figure S4: activation pattern of anxiety in amygdala (AY): anxiety was activated immediately post Agg-E and attenuated 10 and 42 days of post Agg-E.

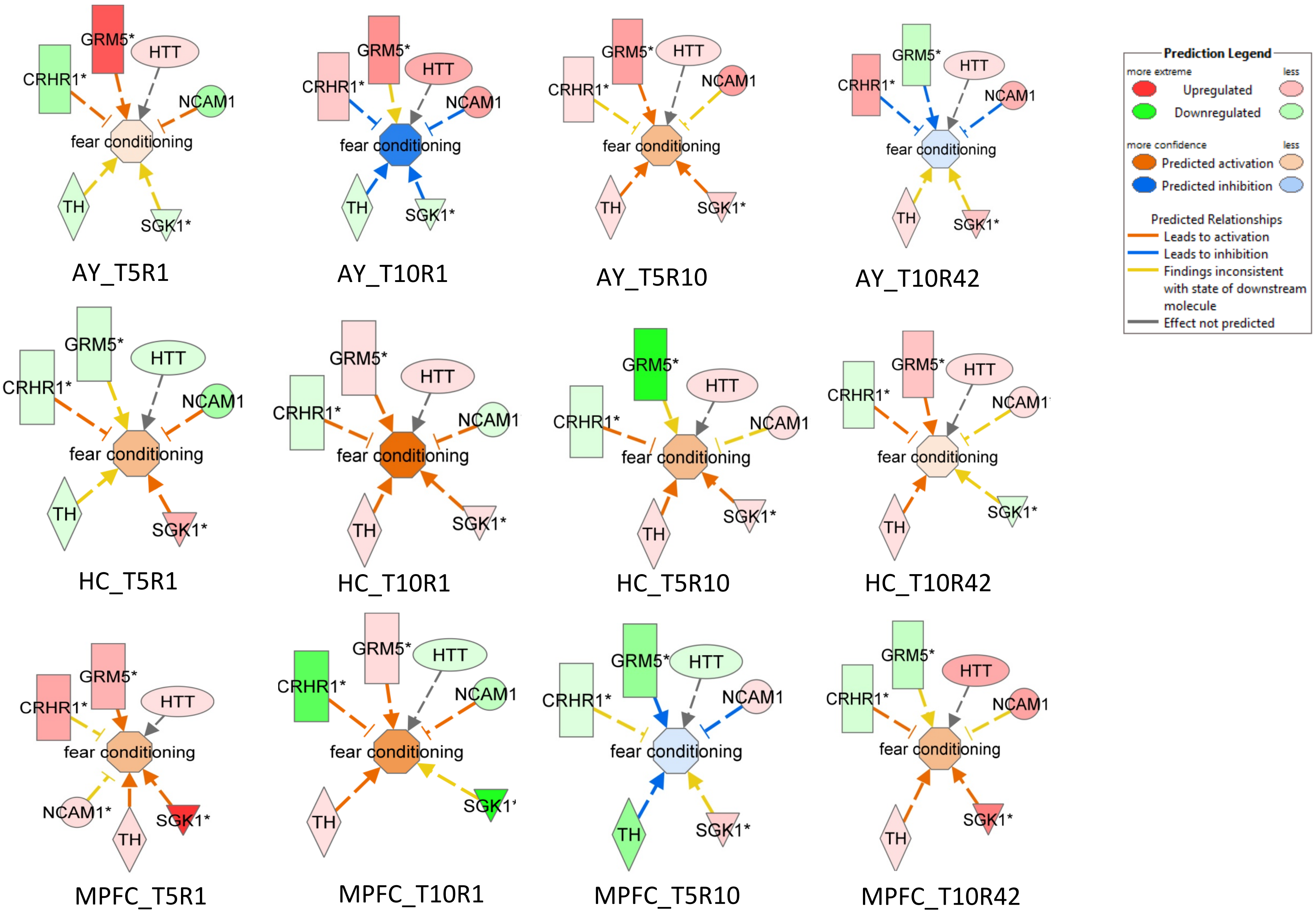

Figure S5: activation pattern of fear conditions in amygdala (AY), hippocampus (HC) and medial prefrontal cortex (MPFC)

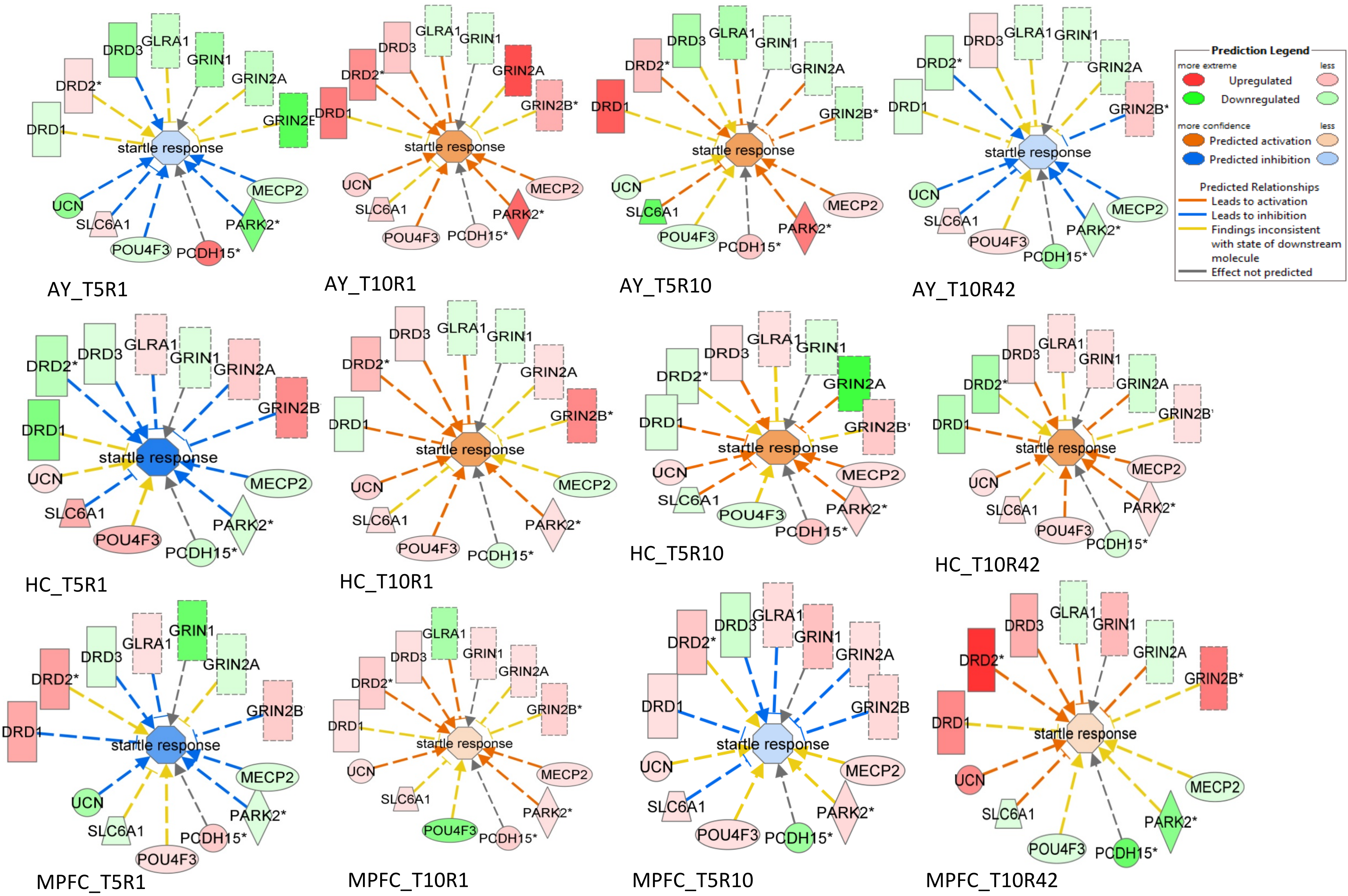

Figure S6: activation pattern of startle response in amygdala (AY), hippocampus (HC) and medial prefrontal cortex (MPFC)

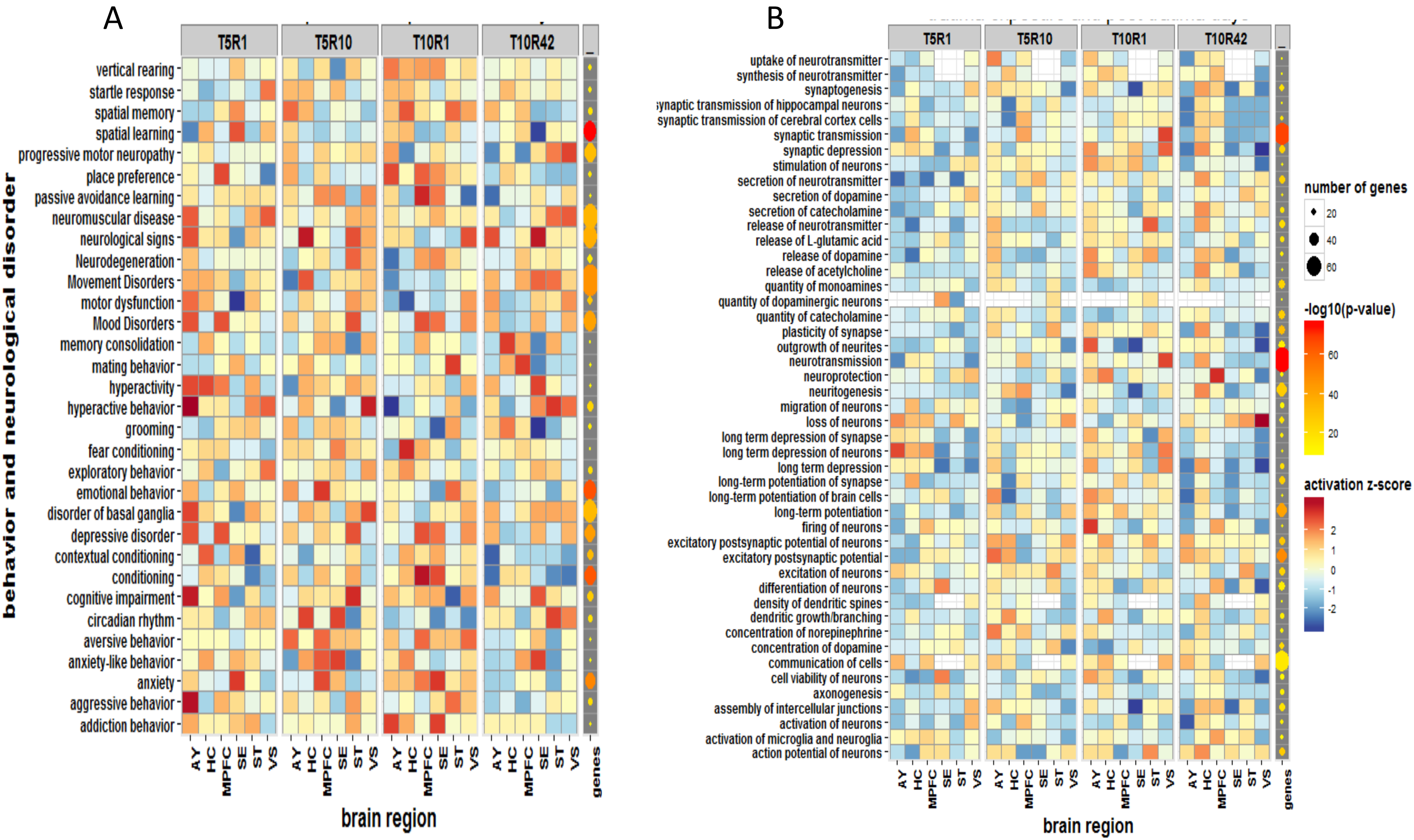

Figure S7: Comparison of activation statuses between behavioral responses (A), and neuronal signaling and synaptic plasticity (B)

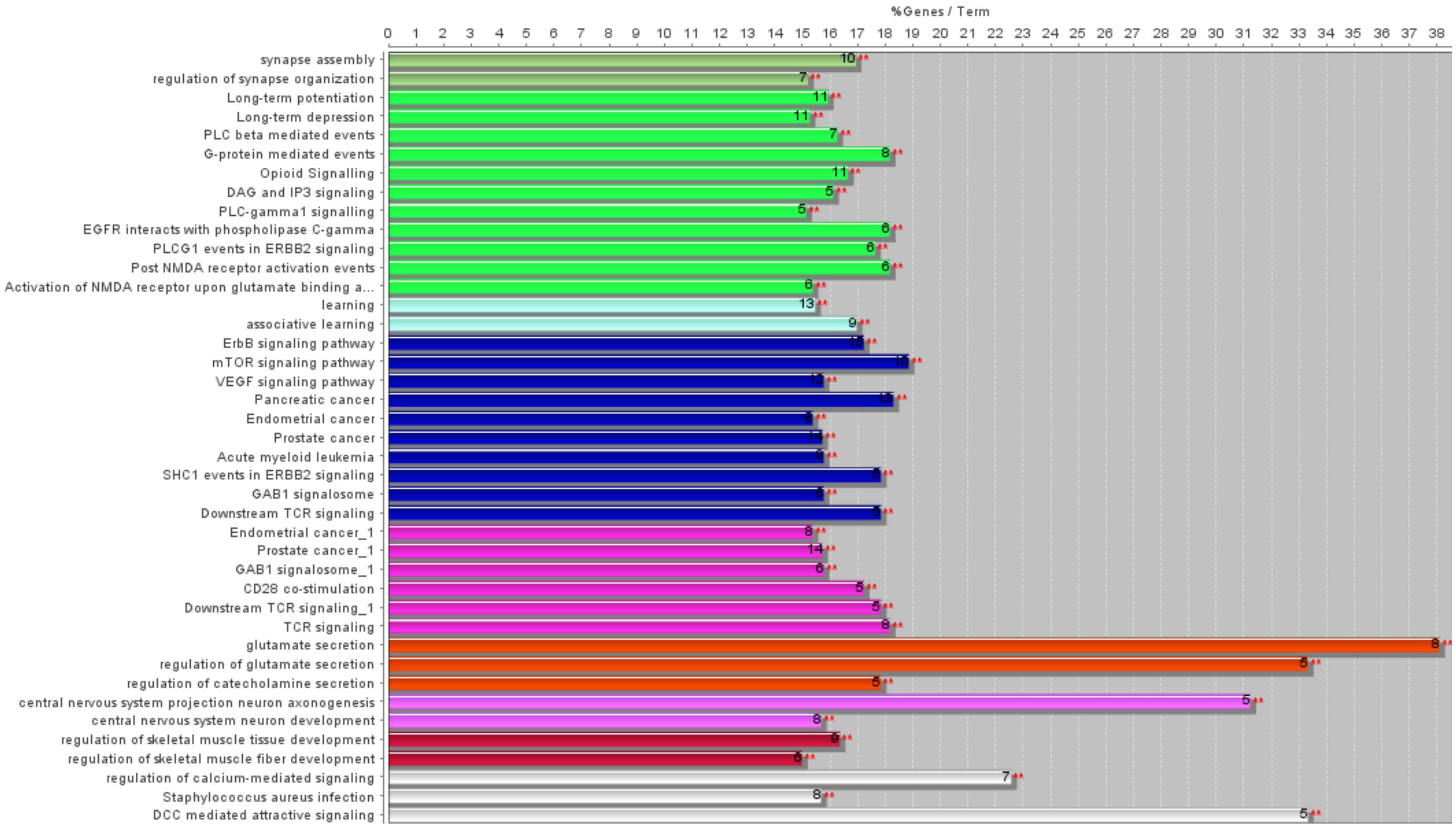

Figure S8: Functions and pathways significantly associated the 306 common DEGs across different time points (and in different brain regions); numbers on at the end of the bars indicate the number of genes associated with the corresponding term.

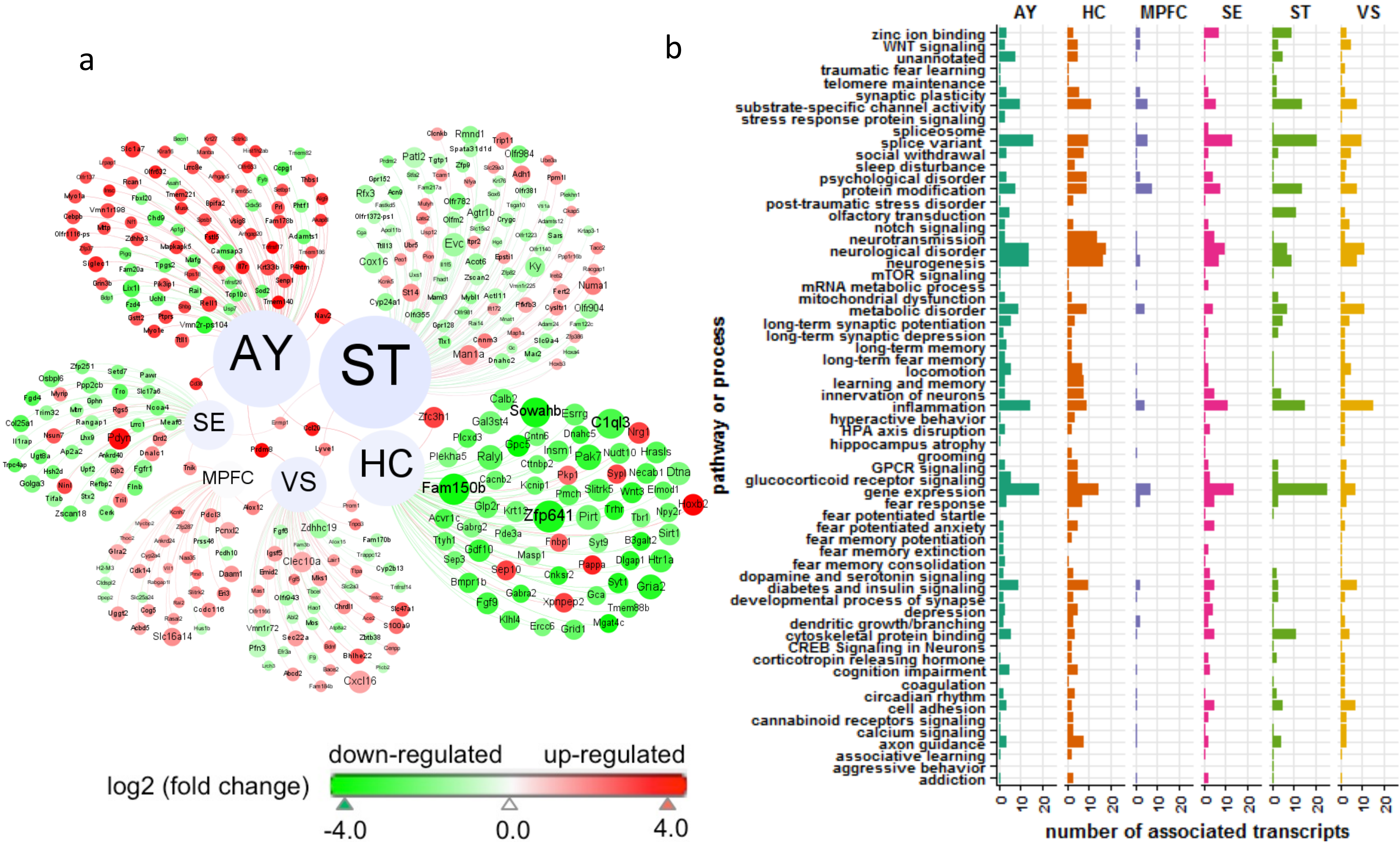

Figure S9: a. Differentially expressed transcripts (p < 0.01 and fold change > 2.0) at 5d1d across brain regions ; b. Significantly associated pathways and biological processes across brain region at 5d1d (associated with transcripts shown in Fig S9.a). Length of the bars are proportional to the numbers of associated transcripts.

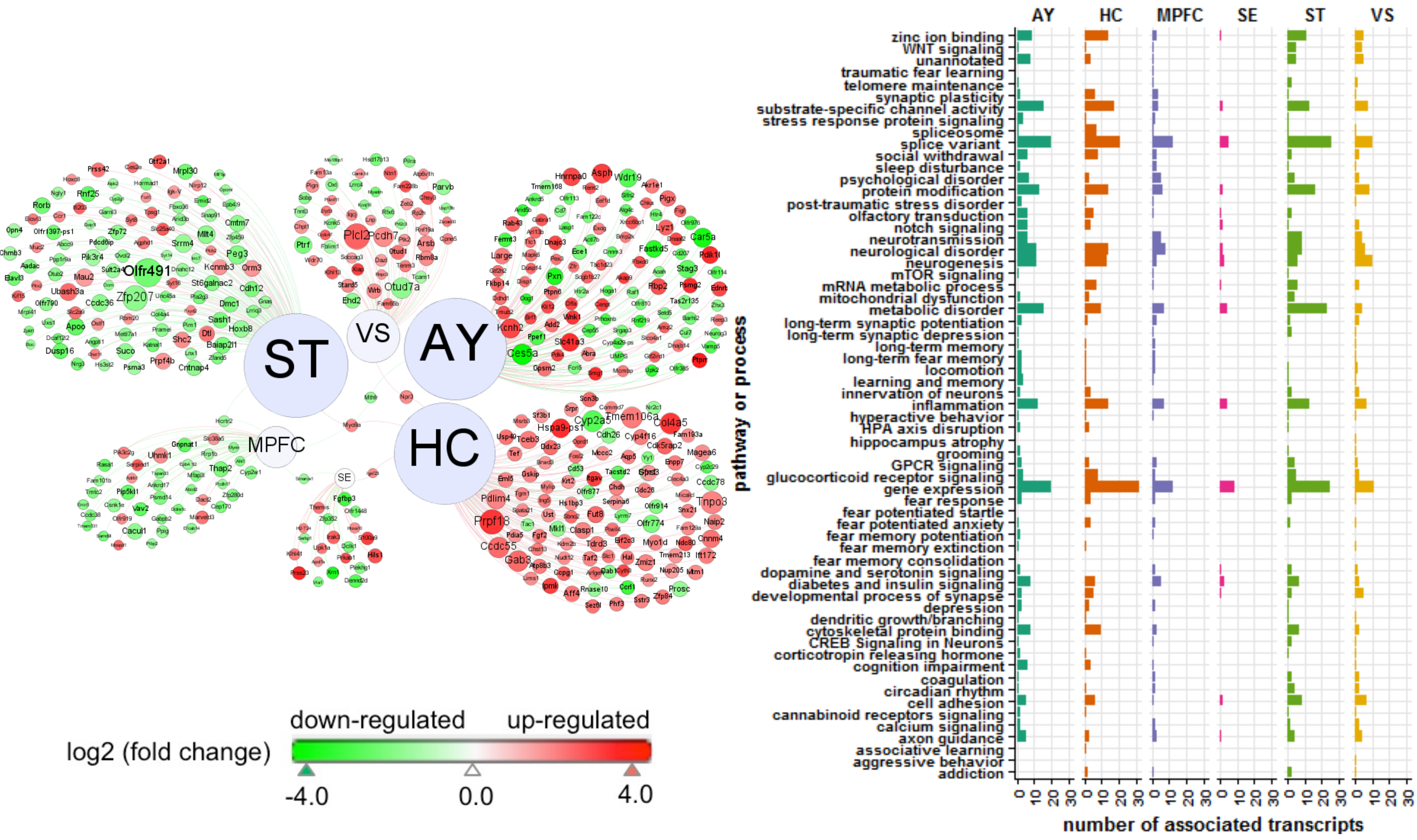

Figure S10: a. Differentially expressed transcripts ($p < 0.01$ and fold change > 2.0) at 5d10d across brain regions; b. Significantly associated pathways and biological processes across brain region at 5d10d (associated with transcripts shown in Fig S10.a). Length of the bars are proportional to the numbers of associated transcripts.

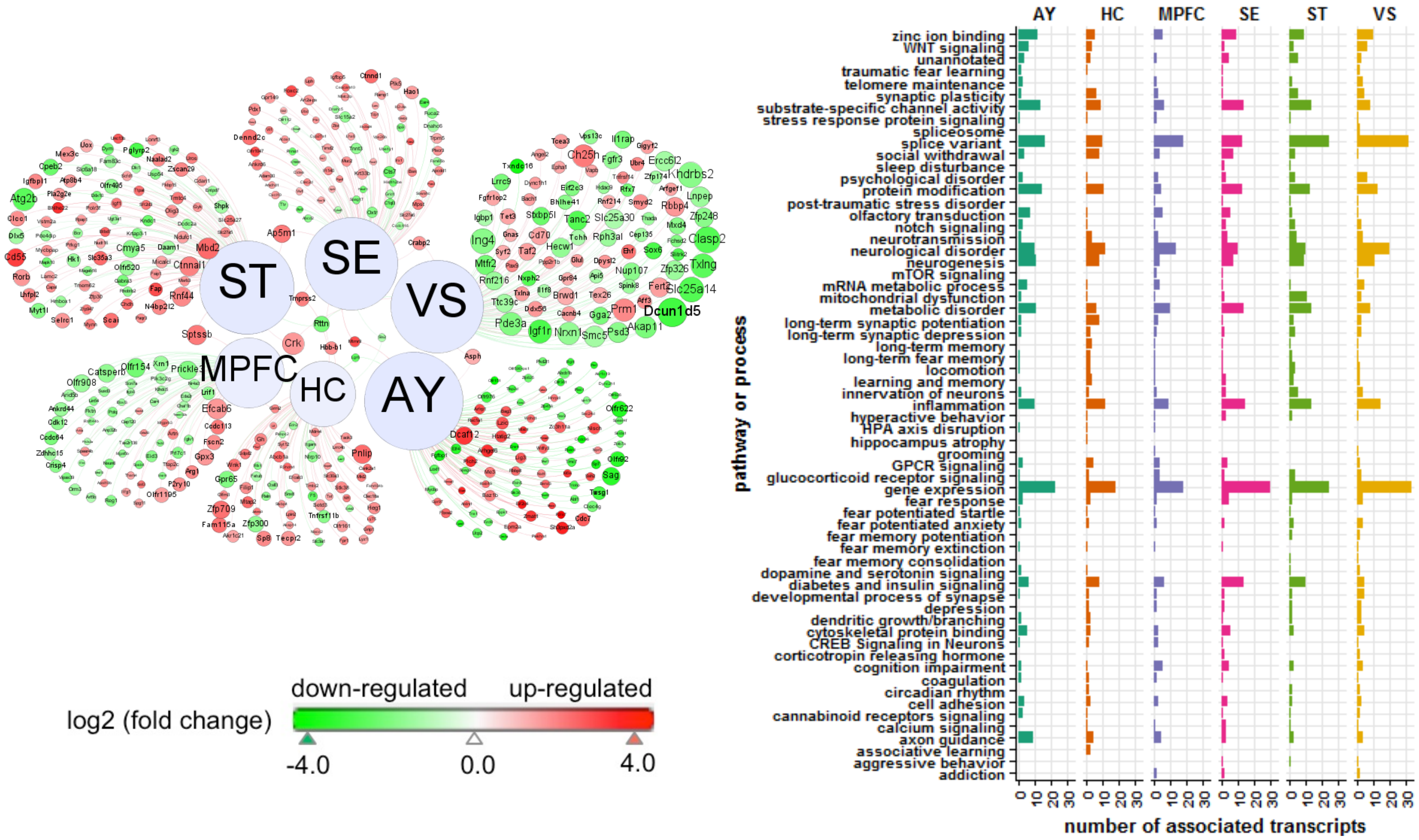

Figure S11: a. Differentially expressed transcripts (p < 0.01 and fold change > 2.0) at 10d1d across brain regions; b. Significantly associated pathways and biological processes across brain region at 10d1d (associated with transcripts shown in Fig S11.a) . Length of the bars are proportional to the numbers of associated transcripts.

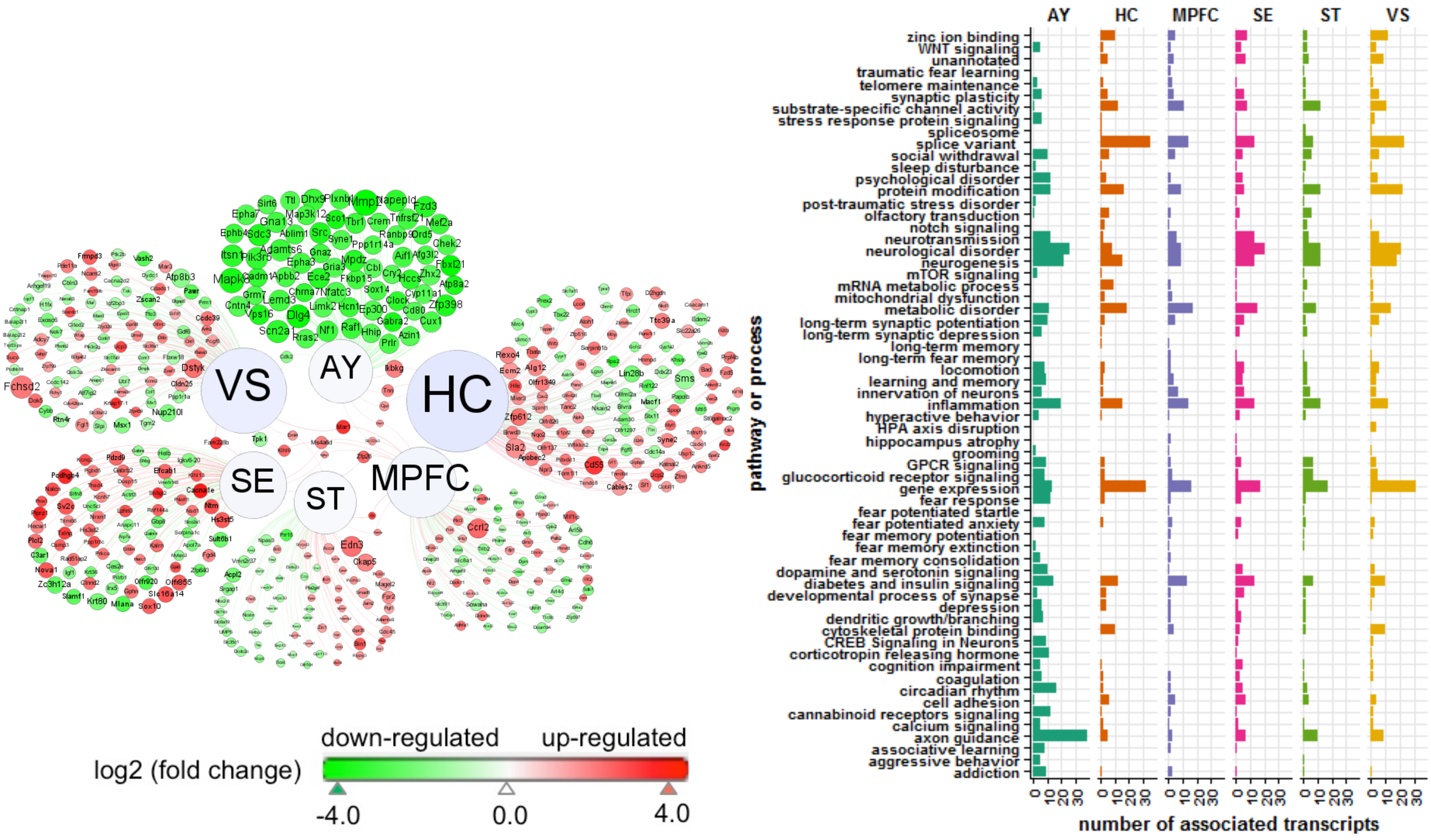

Figure S12: a. Differentially expressed transcripts (p < 0.01 and fold change > 2.0) at 10d42d across brain regions; b. Significantly associated pathways and biological processes across brain region at 10d42d (associated with transcripts shown in Fig S12.a). Length of the bars are proportional to the numbers of associated transcripts.

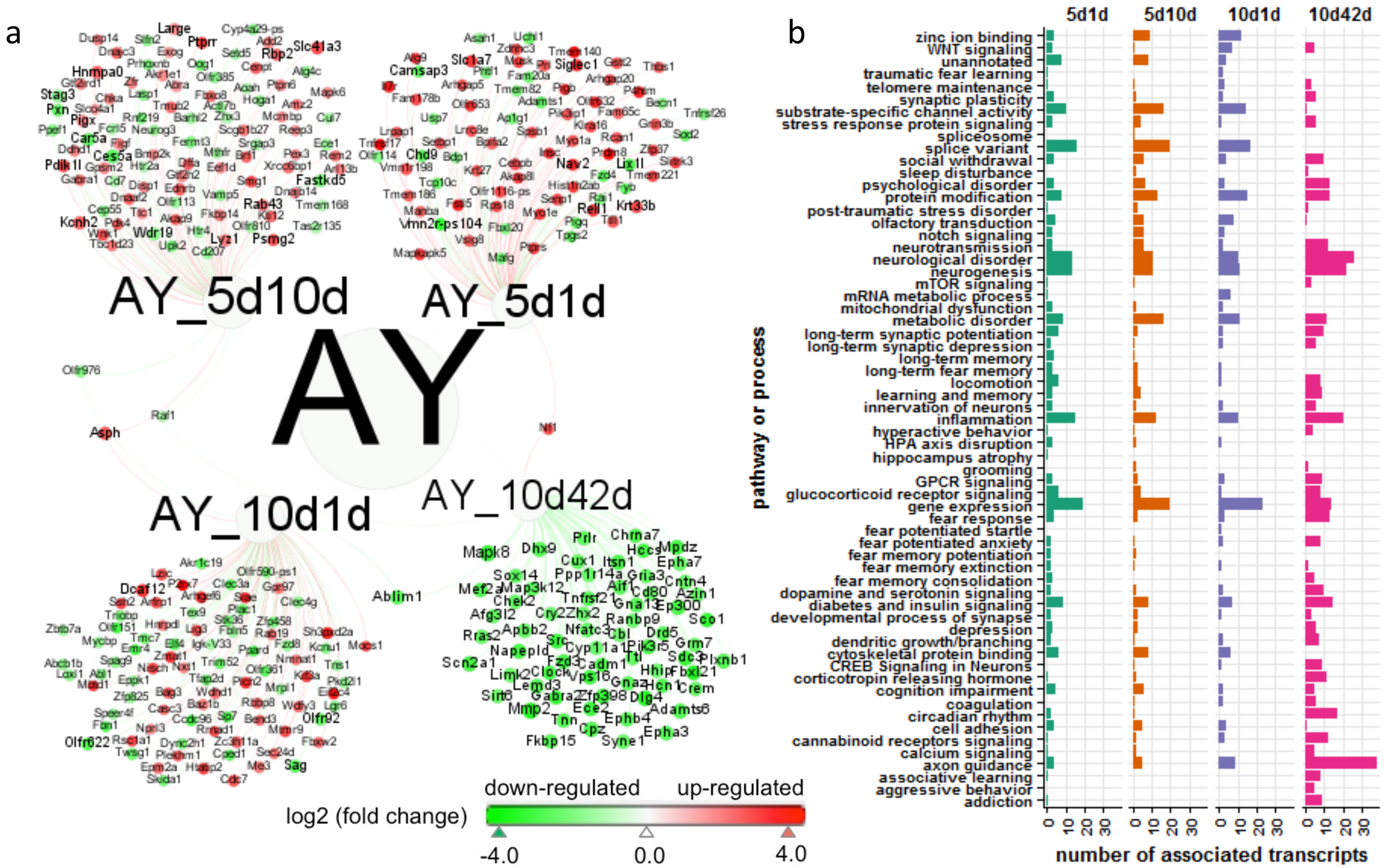

Figure S13: a. differentially regulated transcripts (DEGs: p < 0.01, fold change > 2.0) in amygdala at different time points; and b. pathways or processes associated with DEGs at each time point

Most of the DEGs at 10d42d were down regulated and were associated mainly with axon guidance, circadian rhythm, corticotropin releasing hormone, fear response, addiction, and dopamineregic and serotonergic signalings. On the other hand, splice variant, zinc ion binding, metabolism or gene expression and signal transduction processes were associated with genes at 5d1d, 5d10d and 10d1d time points but not at 10d42d. (Font size is proportional to associated transcripts)

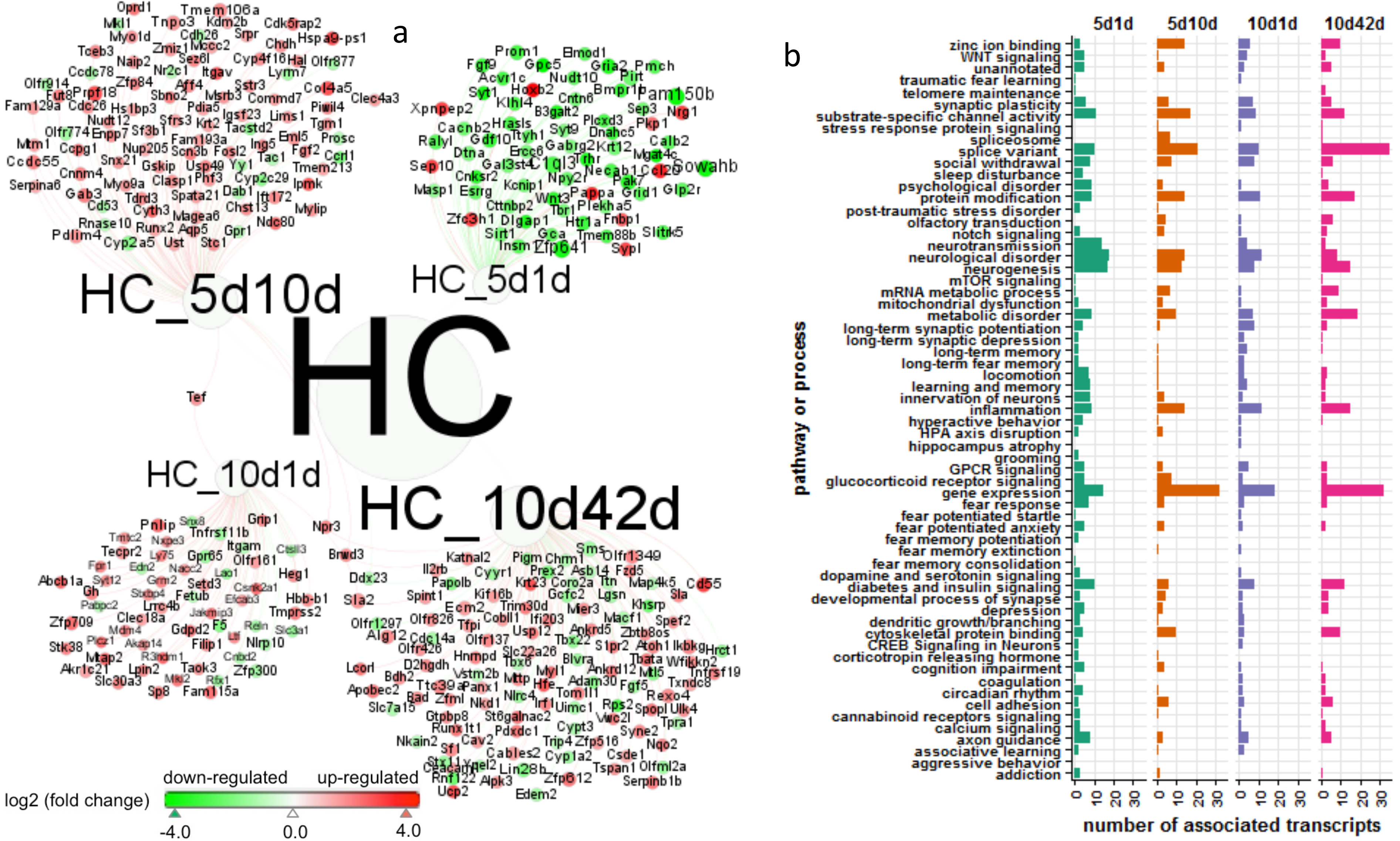

Figure 14: a. differentially regulated transcripts (DEGs: p < 0.01, fold change > 2.0) in Hippocampus (HC) at different time points; and b. pathways or processes associated with DEGs at each time point

Most of the DEGs at 5d1d were down regulated and were associated mainly with axon guidance, nerve impulse, circadian rhythm and fear potentiated anxiety. Splice variant, cation binding, ATP binding, signal transduction and cytoskeletal protein binding were associated with DEGs across time points in HC. But metabolism and gene expression were minimal at 5d1d. (Font size is proportional to associated transcripts)

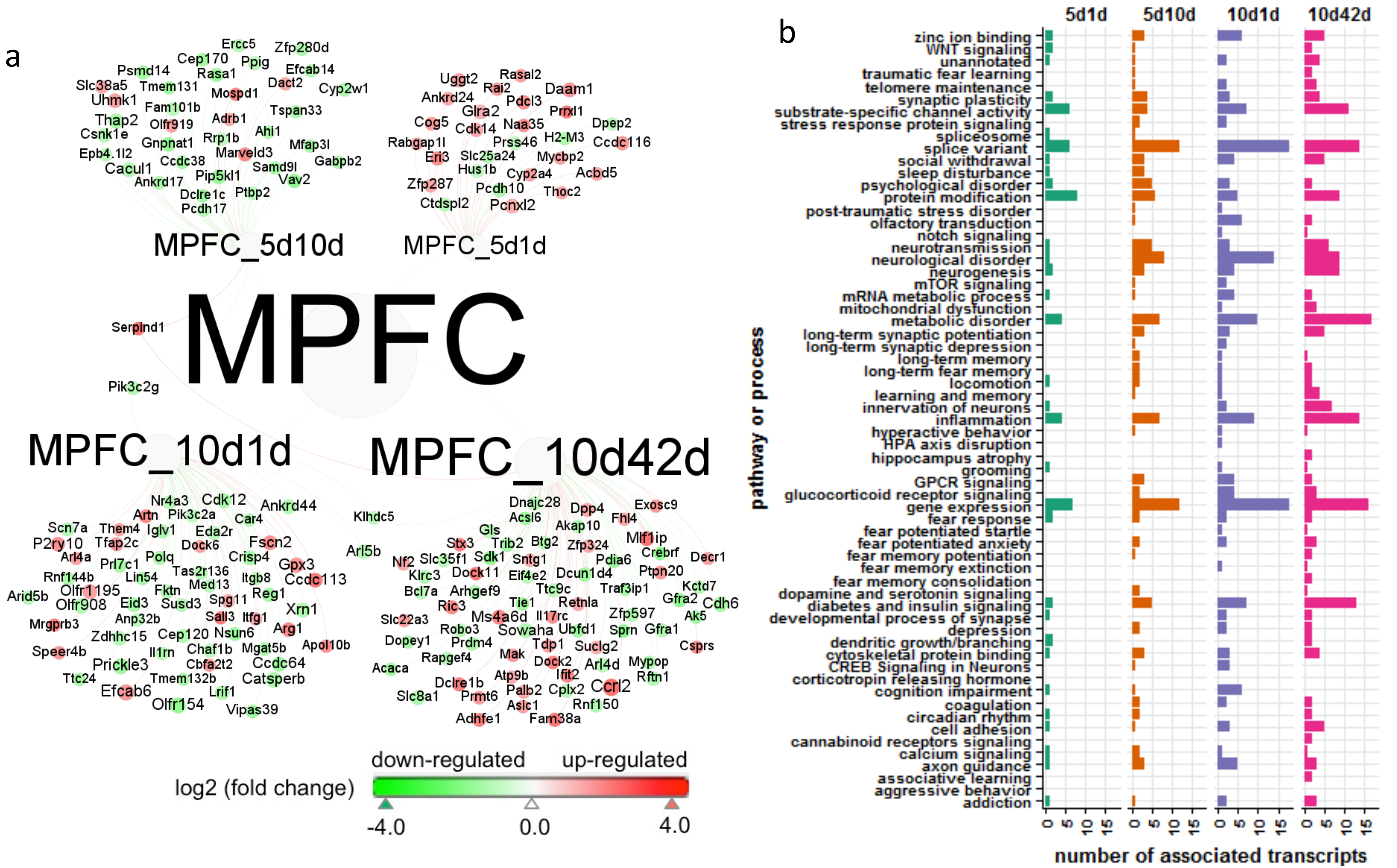

Figure 15: a. differentially regulated transcripts (DEGs: p < 0.01, fold change > 2.0) in medial prefrontal cortex (MPFC) at different time points; and b. pathways or processes associated with DEGs at each time point

Most of the DEGs at 10d42d were associated with wide range of pathways and process compared to other time points (5d1d, 5d10d and 10d1d). But many of the highly represented pathways showed overlap across time points (except some such as ligand receptor activity, which is associated with DEGs at 10d42d). (Font size is proportional to associated transcripts)

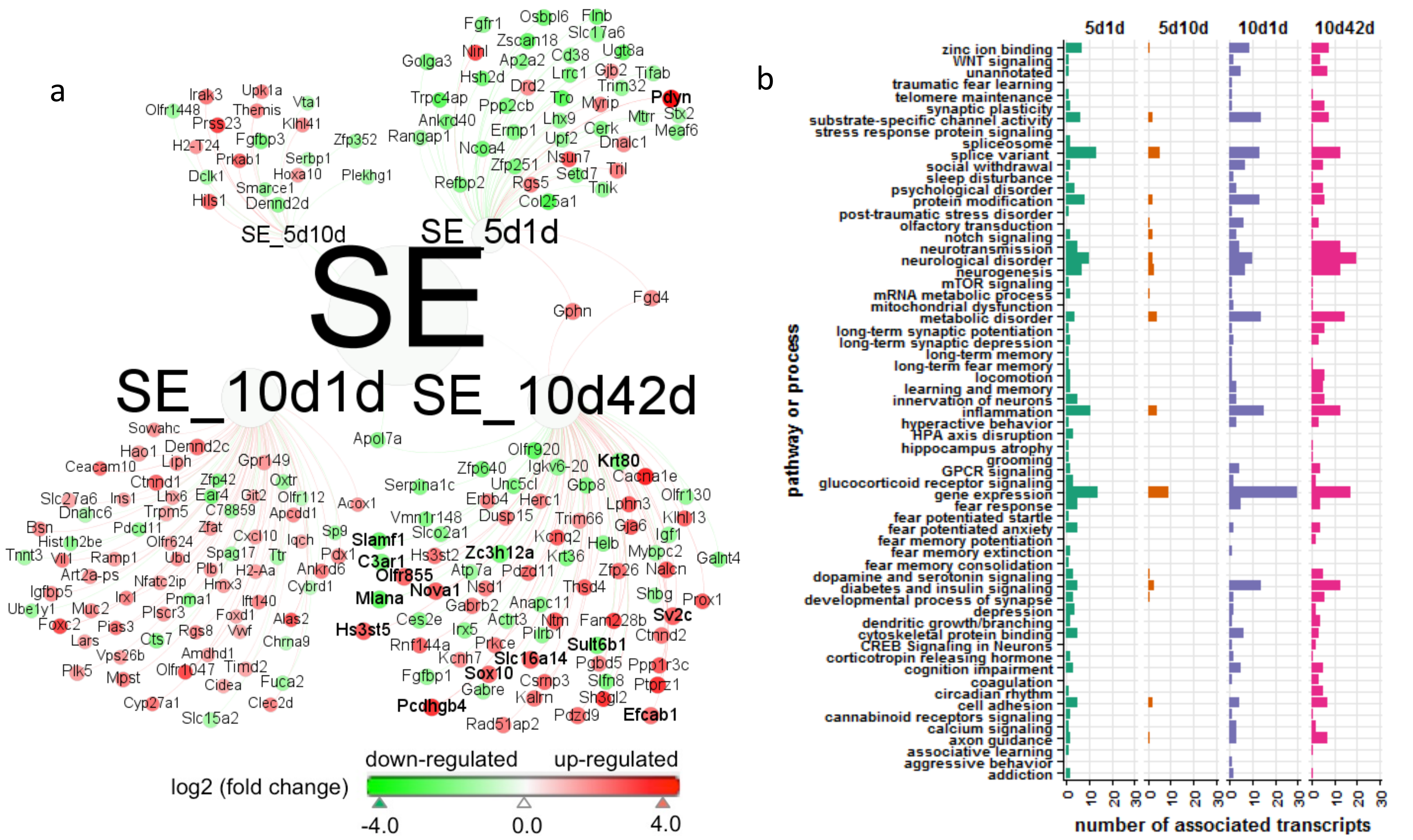

Figure S16: a. differentially regulated transcripts (DEGs: p < 0.01, fold change > 2.0) in septal region (SE) at different time points; and b. pathways or processes associated with DEGs at each time point.
There were fewer transcripts at 5d10d, and hence fewer pathways were associated with DEGs at 5d10d. Signal transduction, gene expression, zinc ion binding and ATP binding were associated with more DEGs at 10d1d than the other time points. Splice variant and metabolism were consistent with number of DEGs across time points. (Font size is proportional to associated transcripts)

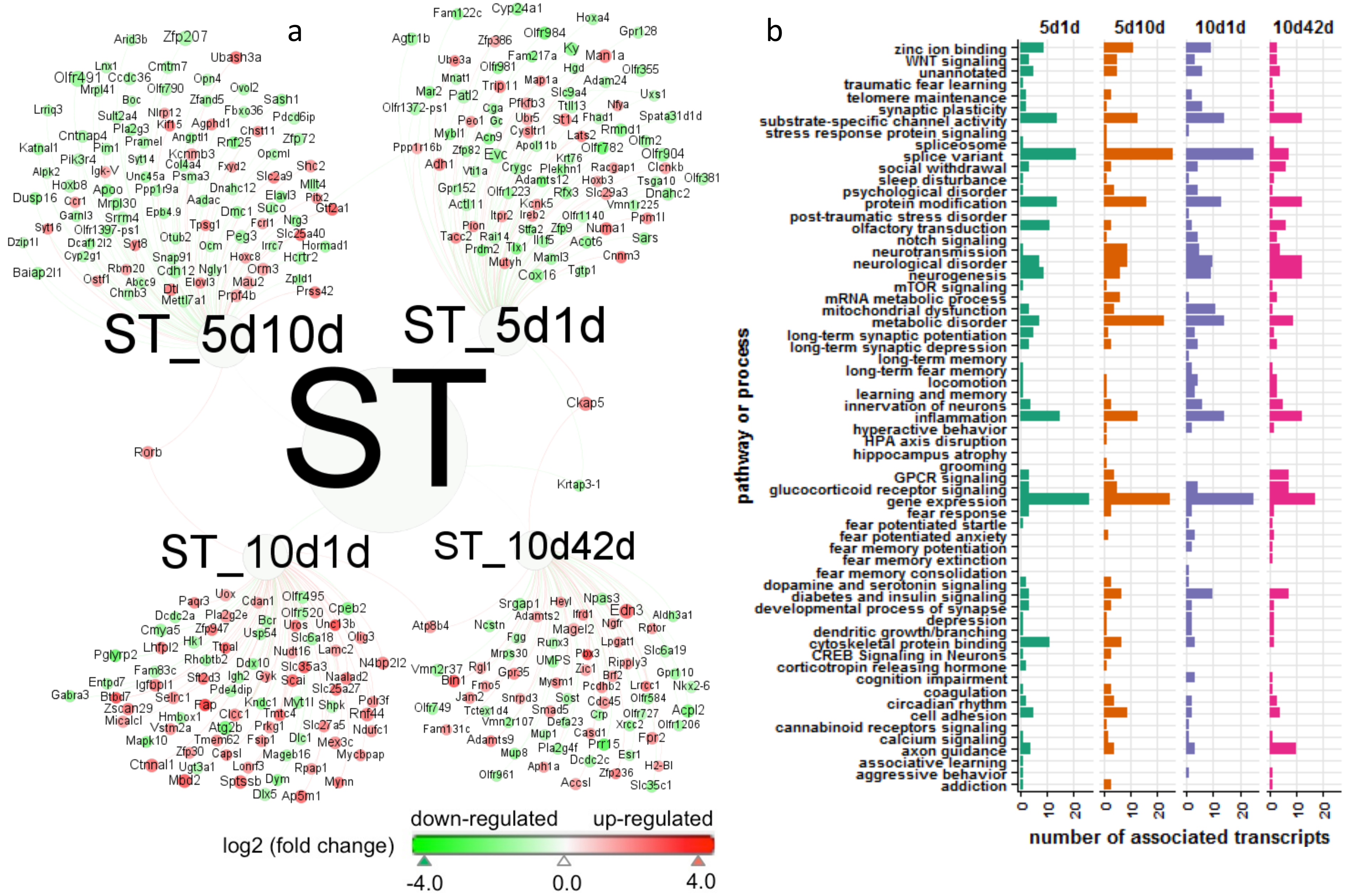

Figure S17: a. differentially regulated transcripts (DEGs: p < 0.01, fold change > 2.0) in corpus striatum (ST) at different time points; and b. pathways or processes associated with DEGs at each time point (Font size is proportional to the number of associated transcripts)

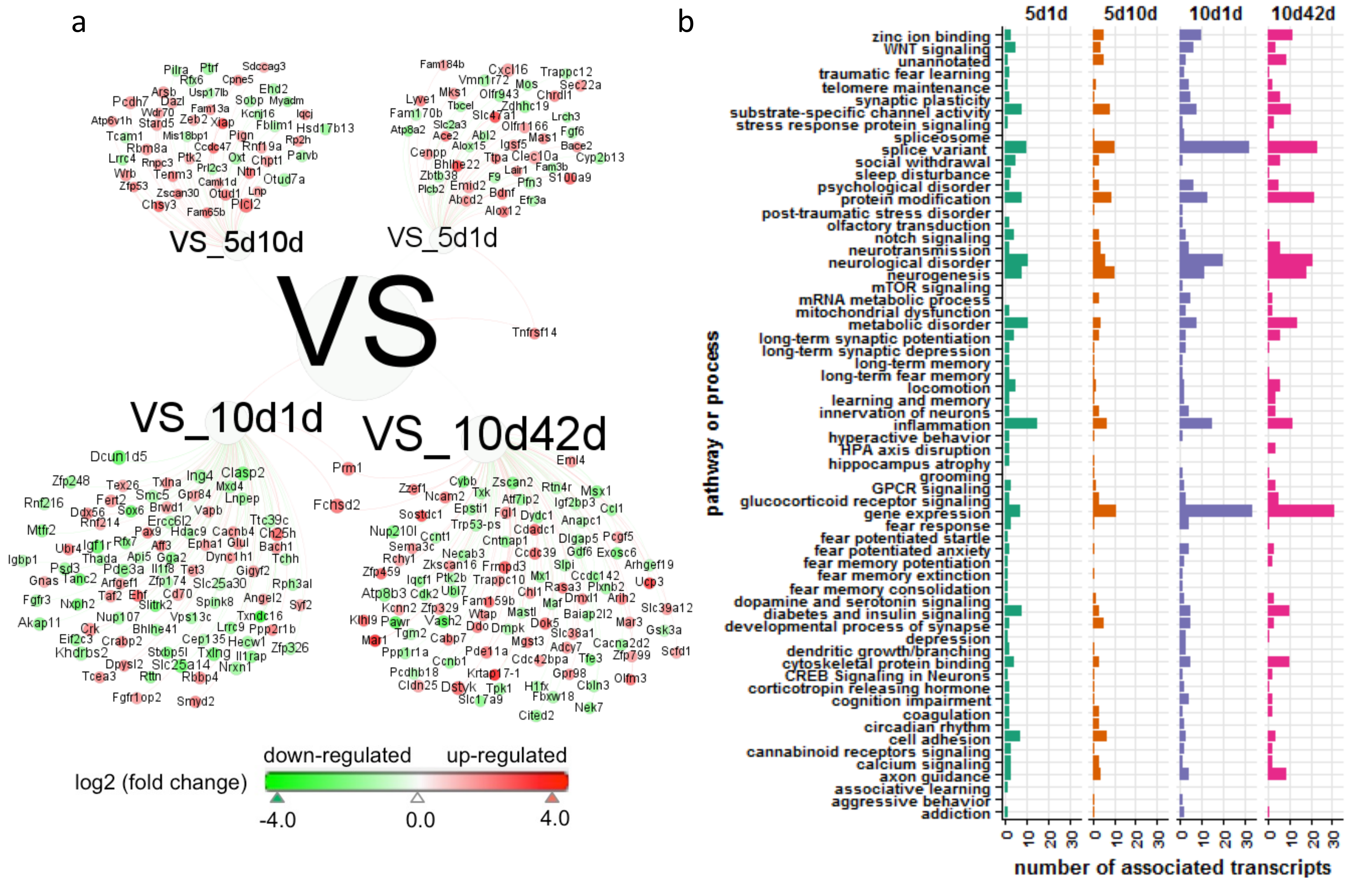

Figure S18: a. differentially regulated transcripts (DEGs: p < 0.01, fold change > 2.0) in ventral striatum (VS) at different time points; and b. pathways or processes associated with DEGs at each time point (Font size is proportional to the number of associated transcripts)

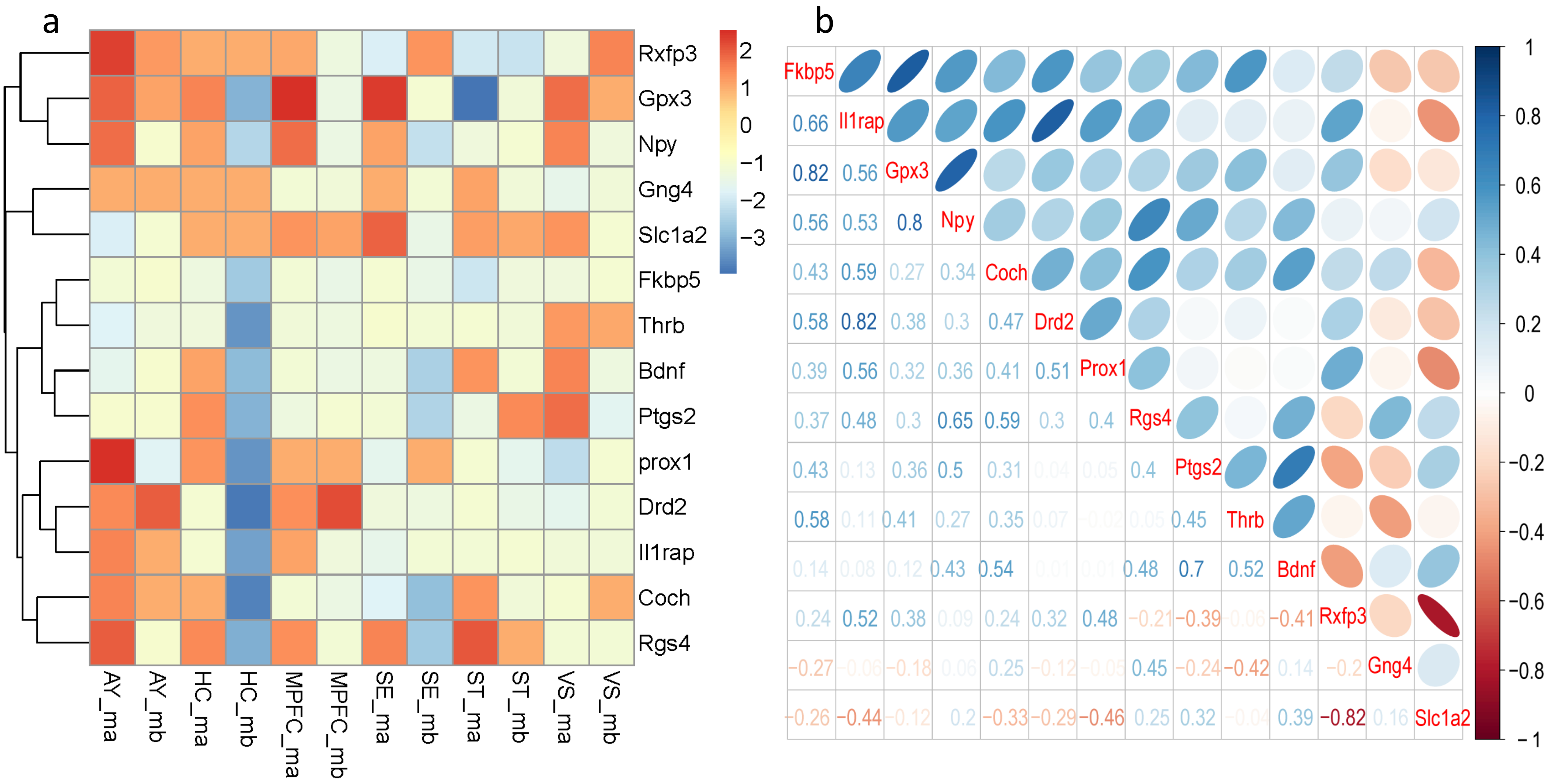

Figure S19: a. Comparison of Quantigene Plex 2.0 Multiplex (bead based) assay and microarray results for PTSD implicated genes in amygdala, hippocampus, medial prefrontal cortex, septum region, corpus striatum and ventral striatum in T10R1 groups (mice exposed for 10 days of social defeat and rested 1 day); ma=microarray and mb=bead based multiplex assay. b. Correlation graph for bead and array results for each transcript, and also correlation matrix for the assayed transcripts (again between the bead and array data). Color palette of the correlation graph shows inter-transcript correlation (blue positive correlation and red negative correlation).

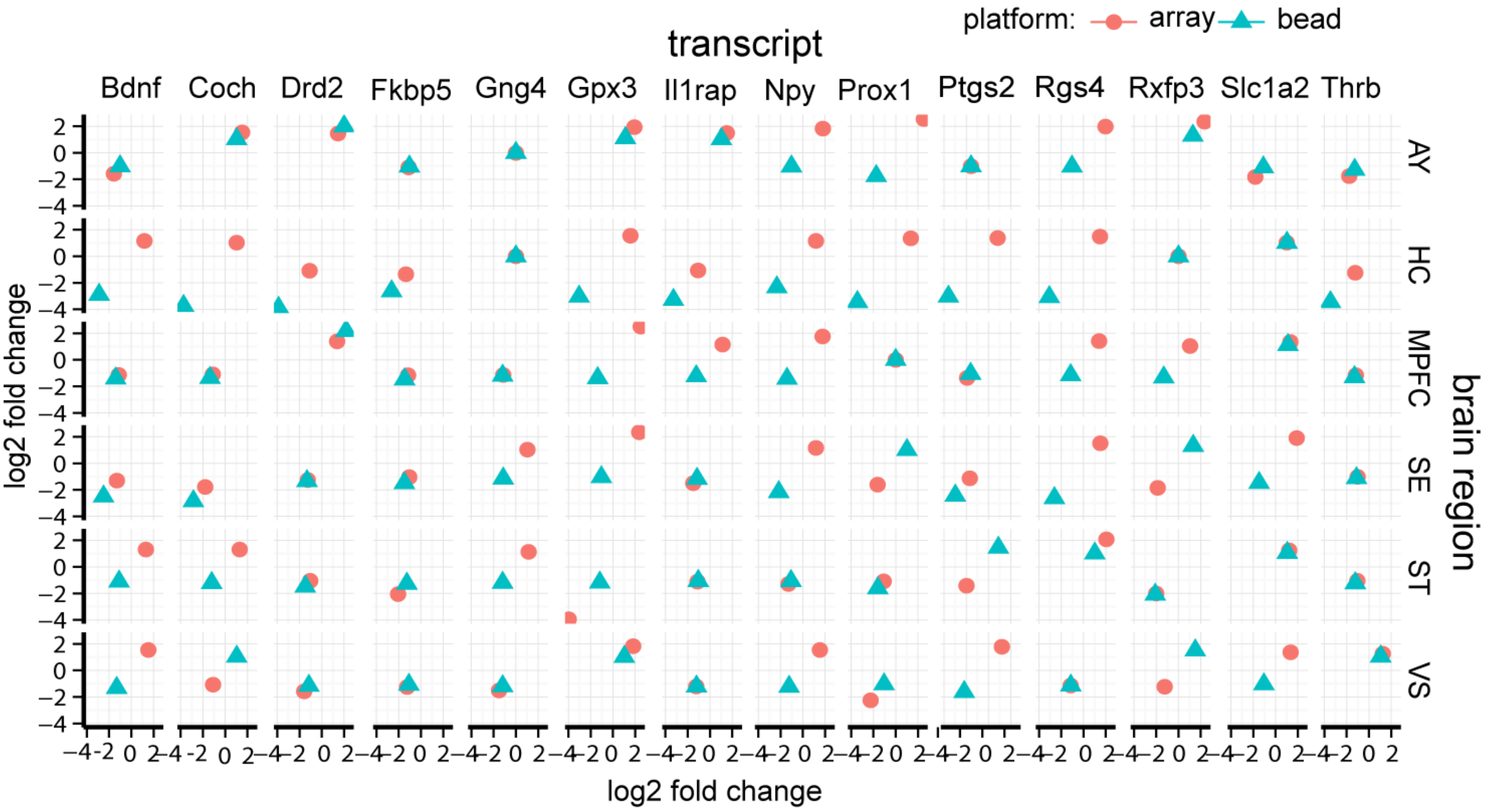

Figure S20: Scatter plot showing comparison of Quantigene Plex 2.0 Multiplex (bead based) assay and microarray results for PTSD implicated genes in amygdala, hippocampus, medial prefrontal cortex, septum region, corpus striatum and ventral striatum in T10R1 groups (mice exposed for 10 days of social defeat and rested 1 day).
